# A Computer Simulation of Progesterone and Cox2 Inhibitor Treatment for Preterm Labor

**DOI:** 10.1371/journal.pone.0008502

**Published:** 2010-01-27

**Authors:** Ozlem Equils, Priya Nambiar, Calvin J. Hobel, Roger Smith, Charles F. Simmons, Shireen Vali

**Affiliations:** 1 Department of Pediatrics, Steven Spielberg Pediatric Research Center, Burns and Allen Research Institute, Cedars-Sinai Medical Center, David Geffen School of Medicine at University of California Los Angeles, Los Angeles, California, United States of America; 2 Medical Division, Pfizer Inc., New York, New York, United States of America; 3 Cellworks Group Inc., Saratoga, California, United States of America; 4 Obstetrics and Gynecology, Cedars-Sinai Medical Center, David Geffen School of Medicine at University of California Los Angeles, Los Angeles, California, United States of America; 5 Endocrine Unit, Mothers and Babies Research Centre, John Hunter Hospital, Hunter Medical Research Institute, Newcastle, New South Wales, Australia; Johns Hopkins University, United States of America

## Abstract

**Background:**

Sufficient information from *in vitro* and *in vivo* studies has become available to permit computer modeling of the processes that occur in the myometrium during labor. This development allows the *in silico* investigation of pathological mechanisms and the trialing of potential treatment*s*.

**Methods/Results:**

Based on the human literature, we developed a computer model of the immune-endocrine environment of the myometrial cell. The interactions between molecules are represented by differential equations. The model is designed to simulate the estrogen and progesterone receptor changes during pregnancy and particularly the changes in the progesterone receptor (PR) isoforms A and B that are thought to mediate functional progesterone withdrawal in the human at labor. Parturition is represented by an increase in the PRA to PRB ratio to levels seen in women in labor. Infection is shown by inducing inflammation in the system by increasing phospho-IkB kinase concentration (IKK) levels; which lead to increased NF-κB activation, causing an increase in the PRA/PRB ratio. We examined the effects of progesterone or cyclo-oxygenase 2 (Cox2) inhibitor treatments on the PRA/PRB ratio *in silico*. The model predicted that high doses of progesterone and Cox2 inhibition would be effective in preventing an NF-κB-induced PRA/PRB ratio increase to the levels found during labor.

**Conclusions:**

Our data illustrate the use of dynamic biological computer simulations to test the effectiveness of therapeutic interventions. This may allow the early rejection of ineffective therapies prior to expensive field trials.

## Introduction

Preterm delivery is a common complication of pregnancy, occurring in approximately 12% of the pregnancies in the US and the rates are increasing. Premature birth is associated with 70% of neonatal mortality and 50% of cases of cerebral palsy [1]. About 45% (>$5 billion/year) of all infant health care expenditure in the US is related to prematurity [1].

Although it is a syndrome rather than a single condition, in >80% of the preterm deliveries <32 weeks of gestation there is intra-uterine infection that is subclinical where maternal and fetal inflammatory response-induced cyclo-oxygenase and placental prostaglandin synthesis lead to myometrial contractility and premature parturition [2].

*In vivo* and *vitro* studies have established that both term and preterm labor are associated with up-regulation of the Cox2 enzyme that synthesizes prostaglandins [3], [4]. Indomethacin, a non-selective cyclooxygenase (Cox) inhibitor has been used widely to prevent preterm delivery; however concerns regarding fetal side effects have limited its use [5]. Recently anecdotal data suggested that Cox2 selective inhibitors may prevent preterm delivery [6].

Progesterone therapy has recently been recommended to prevent preterm delivery in women at high risk women with a previous history of preterm delivery [7] and in those with a short cervix [8]. It is not known if women in whom inflammatory stimuli promote the onset of labor could be treated effectively with progesterone.

There have been no clinical trials of progesterone or Cox2 inhibitor therapy in pregnant women with subclinical infection. We propose that the presence of subclinical infection/inflammation may suppress the therapeutic effect of progesterone and Cox2 inhibitors. Since it is unethical to conduct a prospective experiment without antibiotic treatment of pregnant women with subclinical infection, we developed a computer simulation of the molecular events that lead to an increase in the PRA/PRB ratio to labor levels in the myometrium, and tested the effect of progesterone supplementation and Cox2 specific inhibitors on labor associated changes in biochemical markers as the end-point.

## Results

### NF-κB Activation Leads to an Increase in the PRA/PRB Ratio *In Silico*

We developed a dynamic computer simulation of the molecular events in myometrium at labor. In order to simulate sub-acute and full-blown infection we tested the model at 4 different levels of NF-κB activation. Lowest level NF-κB activation was achieved by a phospho-IkB kinase (IKK) concentration of 0.25 µM (∼80 fold higher than baseline) and this level of NF-κB activation was assumed to be similar to that seen during subclinical-infection. At this level of NF-κB activation there was a limited Cox2 activation (Cox2 mRNA concentration 0.085 mM) and PGE2 protein level was low (0.35 µM = 1.2 mg/ml). The highest level of NF-κB activation was achieved by an IKK concentration of 1 mM. At this level of NF-κB activation Cox2 mRNA concentration was 0.125 mM (1.5 fold increase above baseline) and PGE2 protein concentration was 2.2 µM = 7.7 mg/ml (∼6 fold above baseline).

**Figure 1:**
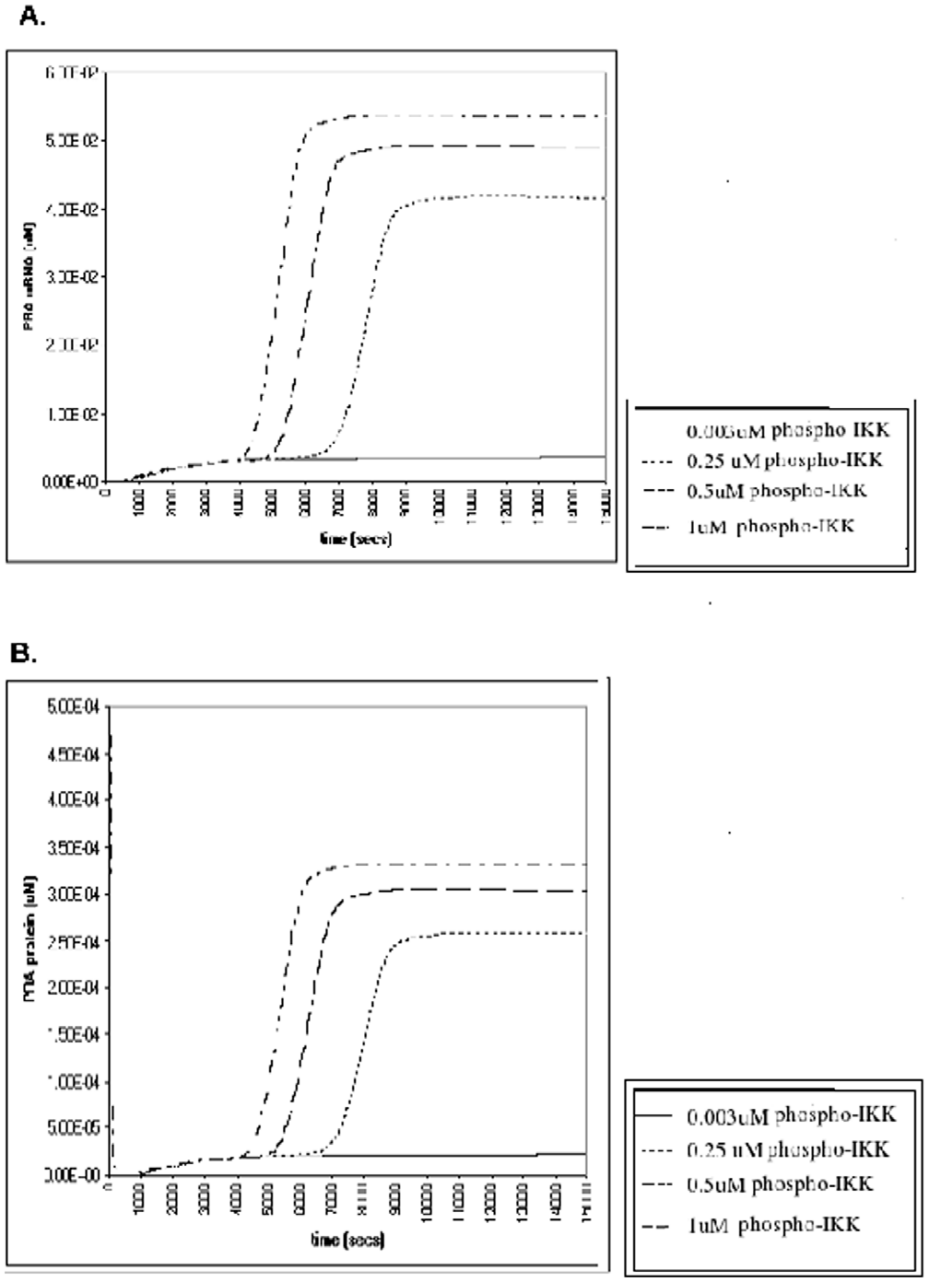
The effect of various levels of NF-κB activation on PRA mRNA and protein expression. We ran the simulation for 15000 seconds. We observed that *in silico* NF-κB activation increased the steady state levels of both PRA mRNA (1A) and protein levels (1B) above those seen in the nonlabor state (0.0033 mM for PRA mRNA and 2.21E-5 µM for PRA protein).

**Figure 2:**
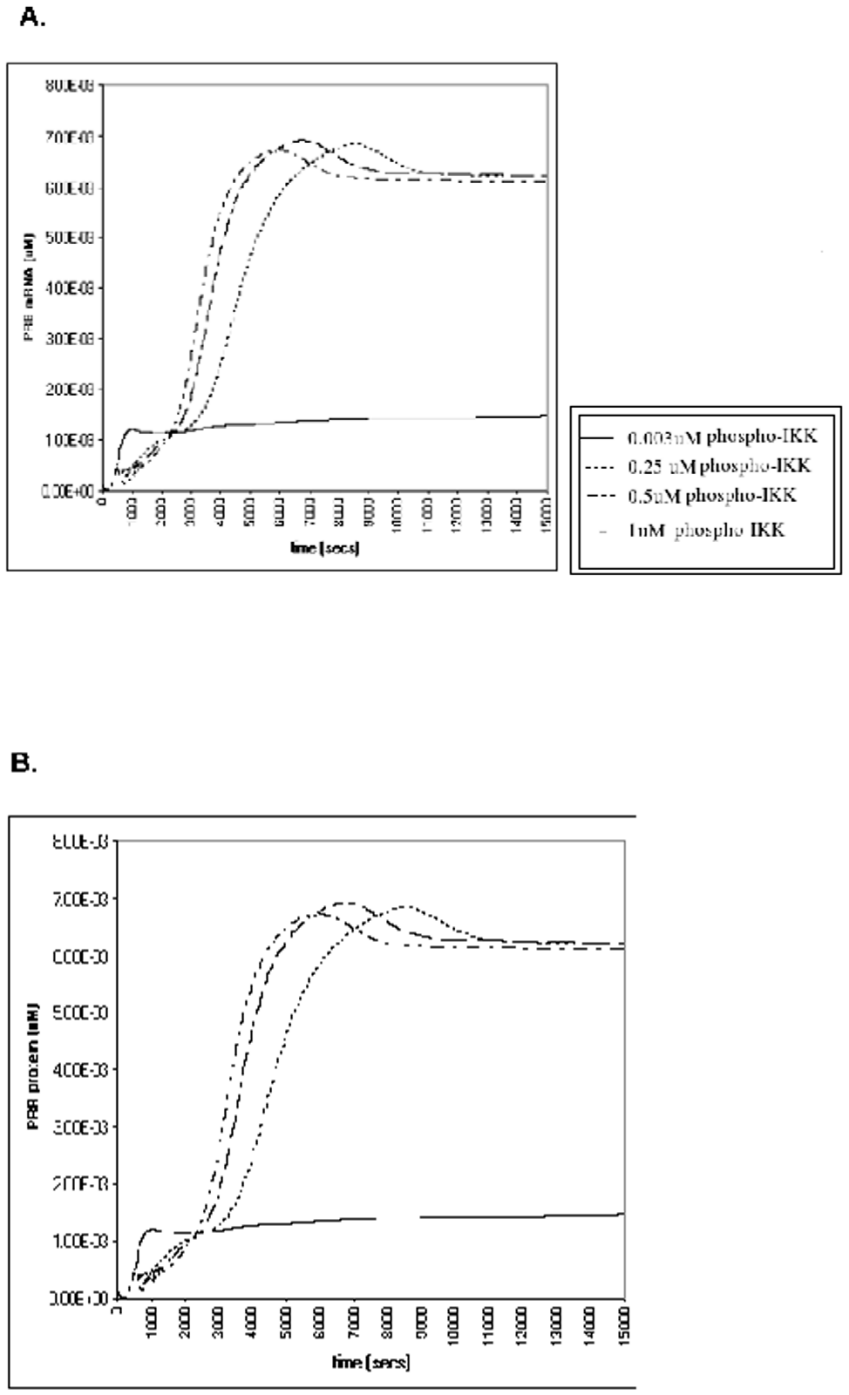
The effect of various levels of NF-κB activation on PRB mRNA and protein expression. We ran the simulation for 15000 seconds. We observed that *in silico* NF-κB activation increased both steady state PRB mRNA (2A) and protein levels (2B) above those seen in the nonlabor state (0.0014 mM for PRB mRNA and 8.89E-6 µM for PRB protein).

**Figure 3:**
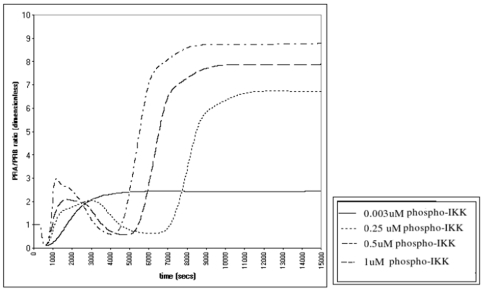
The effect of various levels of NF-κB activation on PRA/PRB. We ran the simulation at various levels of NF-κB activation for 15000 seconds. We observed that *in silico* NF-κB activation increased the PRA/PRB ratio to labor levels (6.2±1.5).

We observed that NF-κB activation led to an increase in PRA and PRB mRNA and protein expression. At lowest level of NF-κB activation PRA protein increased 14 fold above baseline (Figure 1A and 1B); PRB mRNA and protein expression increased 4.2 fold (Figure 2A and 2B respectively). With increasing NF-κB activation PRA and PRB mRNA and protein curves shifted to the left and the PRA/PRB ratio increased to labor levels (6.2±1.5) [9] earlier (Figure 3). At the highest level of NF-κB activation the increase in PRA/PRB ratio was highest (2.9 fold higher than the lowest-nonlabor level of NF-κB activation (Figure 3).

### Progesterone Treatment Has Dose Dependent Effects on NF-κB Induced PRA/PRB Increases

Progesterone treatment has recently been recommended for the prevention of preterm delivery in women with a previous history of preterm delivery and for those with short cervix [7], [8].

**Figure 4:**
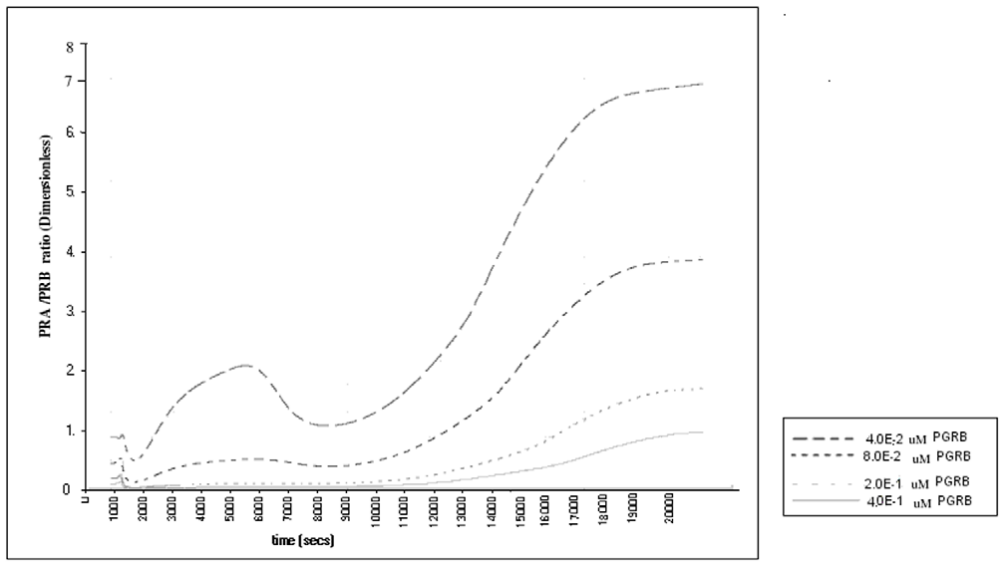
The effect of increased PRB on NF-κB induced PRA/PRB increases. We hypothesized that progesterone treatment effect will be regulated by the level of PRB expression. In the simulation we increased the PRB expression 2, 5 and 10 fold above baseline and observed that only the highest concentration of PRB prevented the PRA/PRB ratio increase to the levels seen at labor (6.2 mean).

We tested the effect of progesterone treatment on the NF-κB-induced PRA/PRB increase. In order to do this we assumed that the protective effect of progesterone would be mediated by PRB [9], [10], and the activity of PRB is determined by the level of tissue PRB expression. Currently there are no data on the concentration of myometrial progesterone receptor expression *in vivo* in pregnant women receiving progesterone supplementation. In order to simulate progesterone treatment we increased the PRB mRNA concentration 2, 5 and 10 fold above the baseline (0.04 µM = 3.96 mg/ml) in the presence of subclinical infection levels of NF-κB activation. We observed that in the presence of subclinical infection the lower two concentrations of progesterone treatment were not effective but at the highest concentration of progesterone, the PRA/PRB ratio was suppressed to pre-parturition levels (Figure 4).

### Cox2 Inhibitors Do Not Prevent Infection Induced Progesterone Withdrawal *In Silico*

**Figure 5:**
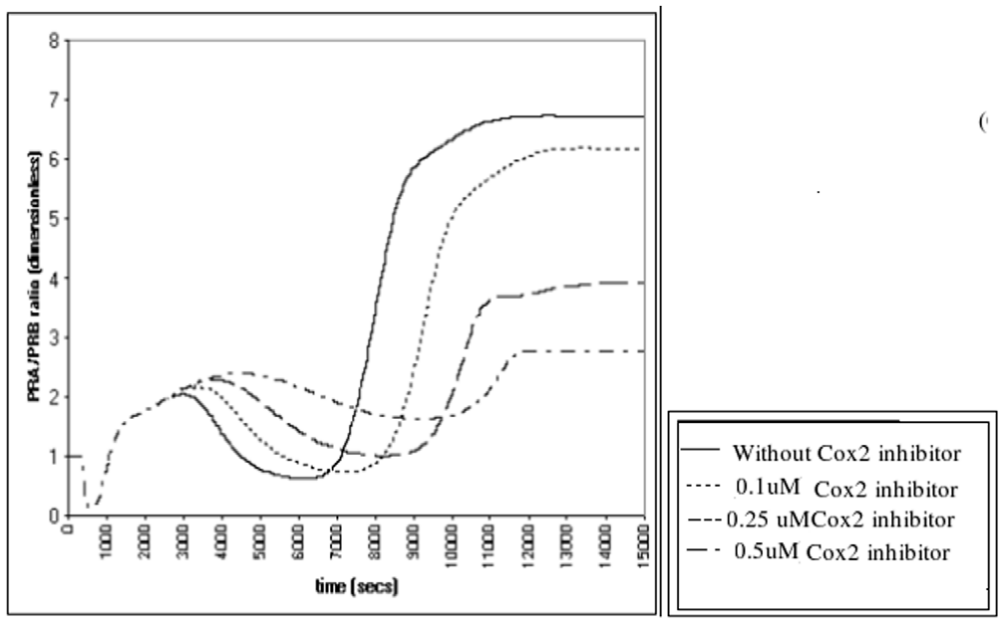
Effect of Cox2 inhibitor on the NF-κB induced PRA/PRB increase. We added a hypothetical Cox2 inhibitor to the system at concentrations of 0.1, 0.25 and 0.5 µM. Only the highest concentration Cox2 inhibitor decreased the PRA/PRB ratio.

Next we tested whether addition of a Cox2 specific inhibitor would prevent the NF-κB-induced PRA/PRB increase. A hypothetical Cox2 inhibitor with Ki (dissociation constant) of 0.051 mM [11], [12] was added at concentrations of 0.1 µM, 0.25 mM and 0.5 µM to the model set at a low level of NF-κB activation. We observed that only the highest concentration of Cox2 inhibitor (0.5 µM) prevented the NF-κB-induced increase in PRA/PRB ratio to labor levels (Figure 5).

## Discussion

Here we used a systems biology approach to test whether the presence of subclinical infection/inflammation would modulate progesterone or Cox2 inhibitor effect on the generation of a PRA to PRB ratio associated with labor. In order to do this we developed a dynamic computer simulation of myometrium at the molecular level using differential equations. We observed that only at the highest concentrations do progesterone and Cox2 specific inhibitors prevent the NF-κB induced PRA/PRB increase. In the case of progesterone there have been recent clinical data that support this observation [13]. In the case of Cox2 inhibition, high concentrations of Cox2 inhibitor may have increased fetal toxicity [14].

System Biology seeks to use mathematical modeling to integrate currently available genomic, proteomic, *in vitro* and *in vivo* data into functional models of biological systems. An effective model of a complex system has a number of potential benefits, notably it may be possible to use the model to predict the behavior of the system when disturbed by pathology or the response of the system to a therapeutic.

The simulations are especially valuable to answer questions that cannot be readily tested, such as new treatments of preterm delivery. The regulation of human parturition is demonstrably different in many ways from that in other mammals. In particular, in most mammals parturition follows a rapid fall in circulating maternal concentrations of progesterone while in the human circulating progesterone levels show no signs of falling until after removal of the placenta. The consequence of the inter-species differences is that animal studies give only limited insight into the mechanisms of human labor. Experimental studies are also problematic in the setting of human labor for ethical reasons.

In this manuscript we have started to develop a model of the molecular events occurring in the human myometrial cell as it transitions at term from non-laboring to the laboring state. Data was obtained from the literature on the perceived critical variables. In this context the critical factors were considered to be the concentrations of progesterone receptors and estrogen receptors and associated factors. To generate the model a number of explicit assumptions were made where clinical or *in vitro* data were unavailable. These assumptions are described in the Methods section.

The model was designed in a bottom-up fashion. Every change to a molecular species, interaction between two or more species, transportation of a species from one compartment to another, transcription and translation is counted as a reaction. The model includes 199 different molecules, 208 reactions, and 624 kinetic parameters.

The model was designed such that activation of NF-κB led to an increase in PRA/PRB ratio to labor levels, reflecting the observation that infection/inflammation is a well known risk factor for preterm delivery. We have then explored how the model responds to a potential tocolytic in the form of a Cox2 inhibitor or progesterone, in the presence of subclinical infection/inflammation. We observed that neither a 10 fold increase in progesterone receptor nor a 2 fold increase in Cox 2 inhibition were effective in preventing the PRA/PRB increase at levels of NF-κB activation that might occur during subclinical infection. These results parallel a recent double-blind, placebo controlled human trial where treatment with a selective Cox2 inhibitor did not reduce the incidence of early preterm delivery [14].

Here we describe the use of a computer model of pregnancy and labor in the myometrium and show that progesterone and Cox2 inhibitor treatments may not be effective in women with subclinical infection. Our results also suggest that a computer simulation can be used as a novel discovery tool to develop hypotheses and test mechanistic and therapeutic hypotheses before moving into lengthy and costly clinical trials.

## Methods

### Building the Model

**Figure 6:**
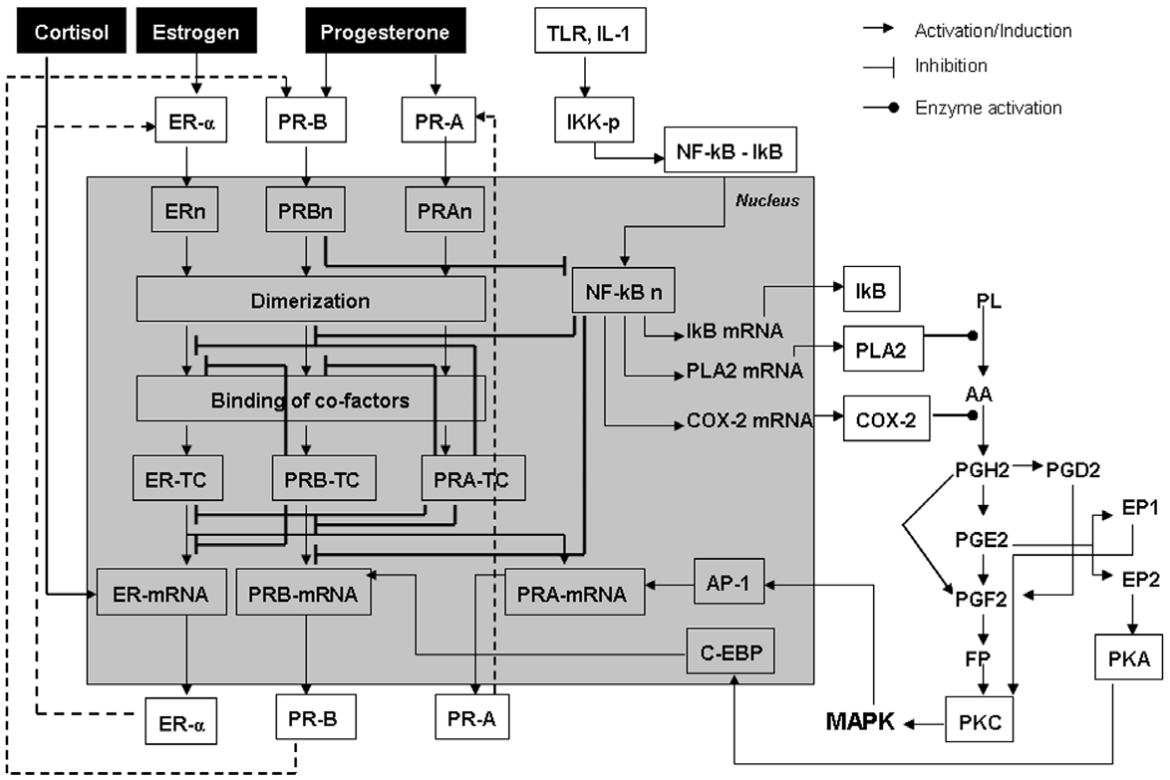
Basic schema of the *in silico* model. The molecular signaling cascades that connect the inflammatory and endocrine environment are shown in a simplified manner in intracellular and intranuclear compartments. PRB-TC – PRB transcription complex; PRA-TC – PRA transcription complex; ER n – ER in nucleus; PRB n – PRB in nucleus; PRA n – PRA in nucleus; NF-κB n – NF-κB in nucleus; FP – FP receptor of prostaglandin (PG)F2; EP1 & EP2 – EP receptors of PGE2; PL – Phospholipid; AA – Arachidonic acid.

First we built a static diagram of the molecular interactions during pregnancy based on Pubmed data (Figure 6). We then created differential equations to express the dynamic interactions between the molecules. The model included three compartments: intracellular, extracellular and intranuclear. Rates of change of the concentration of each molecule with time were modeled as differential equations that were solved by the DOPRI5 method as previously described [15]. The method of development is illustrated with the following examples:

1. Example of an equation representing the irreversible transport of estrogen receptor (ER) mRNA from the nucleus to the cytoplasm:
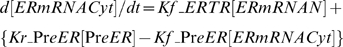

In this equation 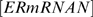 represents nuclear estrogen receptor mRNA concentration,


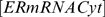
 represents the cytoplasmic estrogen receptor mRNA concentration and 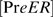 represent the precursor of functional estrogen receptor protein. The above transport reaction has been modeled as a irreversible reaction with 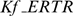 representing the rate of the forward reaction of its formation and its utilization is represented as a reversible reaction with 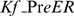 representing the rate of its forward reaction and 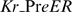 the rate of its reverse reaction.

- Example of an equation representing a binding reaction: Here the change in concentration of cytoplasmic 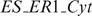 with respect to time would be:
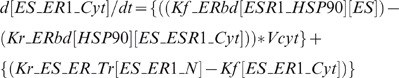

- Binding of estrogen to ER with the release of hsp90 and E2-ER complex.

This is modeled as a reversible reaction. The ligand estrogen represented by ES binds to the estrogen receptor complexes with 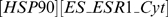 in the cytosol. The binding of estrogen to the receptor causes the release of the Hsp90 chaperone to form the E2_ER complex; which is then translocated to the nucleus.

The model is “run” in a Cellworks Group internal computational engine that solves the differential equations and simulates the dynamic model (please see Supplementary file S1 – JSIM instructions and file S2 - MML file).

### Model Assumptions

#### Progesterone regulation of parturition

During pregnancy there is a continuous supply of progesterone, action of which is controlled by tissue receptor levels [16]. Although multiple progesterone receptors have been described in the myometrium [16], [17], the two major subtypes are PRA and PRB; are encoded by a single gene and are produced independently from separate promoters [18]. PRA is a truncated form of PRB that lacks the first 164 N-terminal amino acids [19]. The PRA promoter has AP-1 binding sites and AP-1 is activated by MAP Kinases which in turn are activated by protein kinase C (PKC); the PRB promoter has a CAAT binding site [20]. In the model we assumed that PRB expression is due to protein kinase A (PKA) induced CAAT/enhancer-binding protein beta (C/EBP) expression; which then binds to the CAAT site of the PRB promoter (Figure 6).

In most human cell types and in the majority of promoter systems examined to date, PRB is the principal ligand-dependent transcriptional activator of progesterone-responsive genes, whereas PRA is a ligand-activated repressor of the transcriptional activity mediated by PRB [19], [21], [22]. *In vitro* studies indicate that PRA blocks progesterone action by inhibiting the transcriptional activity of PRB [20] and consequently suppresses progesterone responsiveness.

Several studies have indicated that in humans parturition is preceded by an increase in the expression of the progesterone dominant negative PRA relative to PRB; which leads to a functional progesterone withdrawal [9], [22]–[25]. In the model the PRA/PRB ratio seen in the myometrium of laboring pregnant women (6.2±1.5) [9] was used as the threshold for parturition. The nonlabor progesterone receptor (PR) B mRNA concentration was set at 0.04 mM and PRA mRNA concentration was set at 0.04 µM based on previous data [9].

### Estrogen Regulation of Parturition

Estrogen is classically considered to be pro-labor. Since there is abundance of estrogen during the pregnancy, the activity of estrogen is thought to be regulated by the tissue estrogen receptor levels. ER alpha is the main receptor in the uterus, expression of which changes during the parturition [9]. We used ER alpha in all simulations and represented as ER. The pre-labor ER mRNA concentration was 0.01 µM in the system.

During pregnancy the transcriptional activity of PRB is higher than that of ER, and PRA transcription increases in association with the onset of labor [22], [24], [26]. ER mediated transcription is inhibited by both PRB and PRA, with the PRB inhibition being more significant [27]. Increases in the PRA to PRB ratio leads to increased expression of the estrogen receptor (ER) alpha and subsequent expression of contraction associated proteins such as connexin 43, oxytocin receptor and cyclo-oxygenase 2 which lead to the development of the synchronous powerful contractions of labor.

#### Coactivators regulate the estrogen and progesterone action

Unliganded PR and ER are kept in the cytoplasm by chaperone proteins (such as Hsp70, Hsp90 and immunophilins), and coactivator molecules, such as CBP, p300 or SRC-1 (steroid receptor coactivator-1) mediate the transcriptional activity of ER and PR by means of their histone acetyl transferase functions. We included the chaperone proteins Hsp70, Hsp90 and immunophilins, and coactivator molecules CBP, p300 and SRC-1 and their dynamic interactions with PR and ER in our model (Figure 6). The model incorporates functional progesterone withdrawal by both altering progesterone receptor (PR) isoform abundance [28] and by diminishing the PRB interaction with progesterone response elements and coregulators due to competition with PRA for these regulators [28], [29].

#### NF-κB is activated during labor

NF-κB is known to play a role in parturition and is activated in amnion cells and myometrial homogenates during parturition [30]. The active NF-κB levels are higher during labor than nonlabor [3], [31]. Currently there are no data on the concentration of phospho-IKK or activated NF-κB in the laboring myometrium *in vivo*. However it has been previously suggested that a small concentration of active NF-κB will be present in all living cells [32]. To show this, we assumed phospho-IKK concentration to be 0.003 µM at baseline; which leads to a steady state NF-κB concentration of 0.0125 µM, compatible with the literature [32]. We assumed phospho-IKK concentration to be 0.5 mM and NF-κB level to be 0.5 mM (∼160 fold higher than baseline) during parturition. This level of NF-κB activation leads to an increase in Cox2 mRNA levels from 0.00054 mM at pre-labor to 0.28 µM at labor, and a 15 fold increase in PRA expression and a 4.2 fold increase in PRB expression with labor. These data corroborate the Cox2 activation observed *in vivo* in the laboring women [9].

#### Model of infection

NF-κB is the main transcription factor responsible for infection induced inflammatory response [33]. In the inactive state NF-κB is found in the cytoplasm bound to IkBα. Infection leads to the activation of a cascade of molecules that lead to the activation of IkB kinase (IKK) which then phosphorylates IkBa; phosphorylated IkBa is ubiquinated and degraded [32], leaving active NF-κB free to move into the nucleus to initiate inflammatory gene expression including the expression of Cox2 [33].

Currently there are no data on infection induced NF-κB activation in the human myometrium. However experiments with human uterine myometrial cells suggest that IL-1 induced NF-κB activation leads to proinflammatory cytokine expression in these cells [34], [35]. Animal models of pregnancy show that intrauterine infection leads to myometrial inflammatory cytokine expression; which suggests NF-κB activation [36], [37].

We assumed that infection will lead to IKK phosphorylation and NF-κB activation in the human pregnant myometrium. In the model, the phospho-IKK level was manipulated by the user to increase the active NF-κB levels.

#### NF-κB regulates the PRA/PRB ratio

NF-κB and the progesterone receptor mutually regulate the activity of each other [38]. PRB binds and inhibits NF-κB mediated transcriptional activation in HeLa cell transfection experiments [38]. NF-κB inhibits PRB-transcription complex formation and PRB-induced PRB transcription in HeLa, COS-1 and T47D breast cancer cell lines [39].

Currently there are no data on the effect of NF-κB activation on myometrial PRA, PRB or ER alpha expression. However it is well established that intrauterine infection is associated with preterm parturition and NF-κB is known to be activated during parturition. Therefore in the model we assumed that NF-κB activation leads to an increase in PRA/PRB ratio. This was achieved by NF-κB induction of PRB expression through protein kinase A and CCAAT/enhancer-binding protein (CEBP) and PRA expression indirectly through MAP kinase and AP-1 activation (Figure 6).

#### Cox2 and labor

Cox2 is activated in the myometrium of laboring women [9]. Cox2 then leads to prostaglandin (PG) expression; cervical PGE2 and PGF2 play the central role in cervical remodeling, effacement, uterine contractions and labor [40], and agonists of both prostaglandins are used in the clinic to induce labor.

Based on previous data we assumed that PGF2 induce protein kinase C (PKC) activation through the FP receptor [41]; PKC then activates MAP kinases to regulate AP-1 expression in the myometrial cells [42]. In our model Cox2 induces PGE2 and PGF2 expression which then leads to PKC activation (Figure 6). PKC then activates MAP kinase phosphorylation, AP-1 activation and increases PRA expression (Figure 6).

## Supporting Information

File S1 (0.01 MB PDF)

File S2 (0.07 MB PDF)

## References

[1] Russell RB, Green NS, Steiner CA, Meikle S, Howse JL (2007). Cost of hospitalization for preterm and low birth weight infants in the United States.. Pediatrics.

[2] Romero R, Espinoza J, Gonçalves LF, Kusanovic JP, Friel L, Hassan S (2007). The role of inflammation and infection in preterm birth.. Semin Reprod Med.

[3] Allport VC, Pieber D, Slater DM, Newton R (2001). Human labour is associated with nuclear factor-kappaB activity which mediates cyclo-oxygenase-2 expression and is involved with the 'functional progesterone withdrawal.. Mol Hum Reprod.

[4] Wang H, Hirsch E (2003). Bacterially-induced preterm labor and regulation of prostaglandin-metabolizing enzyme expression in mice: the role of toll-like receptor 4.. Biol Reprod.

[5] Norton ME, Merrill J, Cooper BA, Kuller JA, Clyman RI (1993). Neonatal complications after the administration of indomethacin for preterm labor.. N Engl J Med.

[6] Sawdy RJ, Groom KM, Bennett PR (2004). Experience of the use of nimesulide, a cyclo-oxygenase-2 selective prostaglandin synthesis inhibitor, in the prevention of preterm labour in 44 high-risk cases.. J Obstet Gynaecol.

[7] Thornton JG (2007). Progesterone and preterm labor–still no definite answers.. N Engl J Med.

[8] Fonseca EB, Celik E, Parra M, Singh M, Nicolaides KH (2007). Progesterone and the risk of preterm birth among women with a short cervix.. N Engl J Med.

[9] Mesiano S, Chan EC, Fitter JT, Kwek K, Yeo G (2002). Progesterone withdrawal and estrogen activation in human parturition are coordinated by progesterone receptor A expression in the myometrium.. J Clin Endocrinol Metab.

[10] Conneely OM, Lydon JP (2000). Progesterone receptors in reproduction: functional impact of the A and B isoforms.. Steroids.

[11] Gierse JK, Zhang Y, Hood WF, Walker MC, Trigg JS (2005). Valdecoxib: assessment of cyclooxygenase-2 potency and selectivity.. J Pharmacol Exp Ther.

[12] Ouellet M, Riendeau D, Percival MD (2001). A high level of cyclooxygenase-2 inhibitor selectivity is associated with a reduced interference of platelet cyclooxygenase-1 inactivation by aspirin.. Proc Natl Acad Sci U S A.

[13] Facchinetti F, Dante G, Venturini P, Paganelli S, Volpe A (2008). 17alpha-hydroxy-progesterone effects on cervical proinflammatory agents in women at risk for preterm delivery.. Am J Perinatol.

[14] Groom KM, Shennan AH, Jones BA, Seed P, Bennett PR (2005). TOCOX–a randomised, double-blind, placebo-controlled trial of rofecoxib (a COX-2-specific prostaglandin inhibitor) for the prevention of preterm delivery in women at high risk.. BJOG.

[15] Raichur A, Vali S, Gorin F (2006). Dynamic modeling of alpha-synuclein aggregation for the sporadic and genetic forms of Parkinson's disease.. Neuroscience.

[16] Speroff L, Glass R, Kase N (1989). Clinical Gynecologic Endocrinology and Infertility. 4th edn..

[17] Weiss G (2000). Endocrinology of parturition.. J Clin Endocrinol Metab.

[18] Kastner P, Krust A, Turcotte B, Stropp U, Tora L (1990). Two distinct estrogen-regulated promoters generate transcripts encoding the two functionally different human progesterone receptor forms A and B.. EMBO J.

[19] Giangrande PH, Kimbrel EA, Edwards DP, McDonnell DP (2000). The opposing transcriptional activities of the two isoforms of the human progesterone receptor are due to differential cofactor binding.. Mol Cell Biol.

[20] Madsen G, Zakar T, Ku CY, Sanborn BM, Smith R (2004). Prostaglandins differentially modulate progesterone receptor-A and -B expression in human myometrial cells: evidence for prostaglandin-induced functional progesterone withdrawal.. J Clin Endocrinol Metab.

[21] Vegeto E, Shahbaz MM, Wen DX, Goldman ME, O'Malley BW (1993). Human progesterone receptor A form is a cell- and promoter-specific repressor of human progesterone receptor B function.. Mol Endocrinol.

[22] Tung L, Mohamed MK, Hoeffler JP, Takimoto GS, Horwitz KB (1993). Antagonist-occupied human progesterone B-receptors activate transcription without binding to progesterone response elements and are dominantly inhibited by A-receptors.. Mol Endocrinol.

[23] Mesiano S (2001). Roles of estrogen and progesterone in human parturition.. Front Horm Res.

[24] Pieber D, Allport VC, Hills F, Johnson M, Bennett PR (2001). Interactions between progesterone receptor isoforms in myometrial cells in human labour Mol Hum Reprod.

[25] Pieber D, Allport VC, Bennett PR (2001). Progesterone receptor isoform A inhibits isoform B-mediated transactivation in human amnion.. Eur J Pharmacol.

[26] Bernard A, Duffek L, Torok I, Kosa Z (1988). Progesterone and oestradiol levels and cytoplasmic receptor concentrations in the human myometrium at term, before labour and during labour.. Acta Physiol Hung.

[27] Wen DX, Xu YF, Mais DE, Goldman ME, McDonnell DP (1994). The A and B isoforms of the human progesterone receptor operate through distinct signaling pathways within target cells.. Mol Cell Biol.

[28] Henderson D, Wilson T (2001). Reduced binding of progesterone receptor to its nuclear response element after human labor onset. Am Progesterone receptor isoform A inhibits isoform B-mediated transactivation in human amnion.. J Obstet Gynecol.

[29] Smith R (2007). Parturition.. N Engl J Med.

[30] Chapman NR, Europe-Finner GN, Robson SC (2004). Expression and deoxyribonucleic acid-binding activity of the nuclear factor kappaB family in the human myometrium during pregnancy and labor.. J Clin Endocrinol Metab.

[31] Lindstrom TM, Bennett PR (2005). The role of nuclear factor kappa B in human labour.. Reproduction.

[32] Sung MH, Simon R (2004). In silico simulation of inhibitor drug effects on nuclear factor-kappaB pathway dynamics.. Mol Pharmacol.

[33] Carmody RJ, Chen YH (2007). Nuclear factor-kappaB: activation and regulation during toll-like receptor signaling.. Cell Mol Immunol.

[34] Erkinheimo TL, Saukkonen K, Narko K, Jalkanen J, Ylikorkala O (2000). Expression of cyclooxygenase-2 and prostanoid receptors by human myometrium.. J Clin Endocrinol Metab.

[35] Bartlett SR, Sawdy R, Mann GE (1999). Induction of cyclooxygenase-2 expression in human myometrial smooth muscle cells by interleukin-1beta: involvement of p38 mitogen-activated protein kinase.. J Physiol.

[36] Wang H, Hirsch E (2003). Bacterially-induced preterm labor and regulation of prostaglandin-metabolizing enzyme expression in mice: the role of toll-like receptor 4.. Biol Reprod.

[37] Hirsch E, Filipovich Y, Mahendroo M (2006). Signaling via the type I IL-1 and TNF receptors is necessary for bacterially induced preterm labor in a murine model.. Am J Obstet Gynecol.

[38] van der Burg B, van der Saag PT (1996). Nuclear factor-kappa-B/steroid hormone receptor interactions as a functional basis of anti-inflammatory action of steroids in reproductive organs.. Mol Hum Reprod.

[39] Kalkhoven E, Wissink S, van der Saag PT, van der Burg B (1996). Negative interaction between the RelA(p65) subunit of NF-kappaB and the progesterone receptor.. J Biol Chem.

[40] Khan AH, Carson RJ, Nelson SM (2008). Prostaglandins in labor-a translational approach.. Front Biosci.

[41] Olson DM, Ammann C (2007). Role of the prostaglandins in labour and prostaglandin receptor inhibitors in the prevention of preterm labour.. Front Biosci.

[42] Geimonen E, Jiang W, Ali M, Fishman GI, Garfield RE (1996). Activation of protein kinase C in human uterine smooth muscle induces connexin-43 gene transcription through an AP-1 site in the promoter sequence.. J Biol Chem.

