# Correction: A Computer Simulation of Progesterone and Cox2 Inhibitor Treatment for Preterm Labor

**DOI:** 10.1371/annotation/d3491dea-c206-4660-940e-7fefdcee5761

**Published:** 2010-02-24

**Authors:** Ozlem Equils, Priya Nambiar, Calvin J. Hobel, Roger Smith, Charles F. Simmons, Shireen Vali

The affiliations of the fifth author, Charles F. Simmons, are incorrect. He is affiliated only with #1, Department of Pediatrics, Steven Spielberg Pediatric Research Center, Burns and Allen Research Institute, Cedars-Sinai Medical Center, David Geffen School of Medicine at University of California Los Angeles, Los Angeles, California, United States of America

