# Supplementary material for: A Computer Simulation of Progesterone and Cox2 Inhibitor Treatment for Preterm Labor
Source: PLoS One. 2010 Jan 27;5(1):e8502. doi: 10.1371/journal.pone.0008502 (PMC2811723; doi:10.1371/journal.pone.0008502)
Supplement: File S1 — (0.01 MB PDF) [file pone.0008502.s001.pdf]

Jsim takes mml file as .mod extension and JSim v1.1 needs to be added in the top of the file.

Simple instructions to follow,

./jsim # JSIM gui invocation.

ADD menu - Load .mod file and then compile.That is it.
