# Supplementary material for: A Computer Simulation of Progesterone and Cox2 Inhibitor Treatment for Preterm Labor
Source: PLoS One. 2010 Jan 27;5(1):e8502. doi: 10.1371/journal.pone.0008502 (PMC2811723; doi:10.1371/journal.pone.0008502)
Supplement: File S2 — (0.07 MB PDF) [file pone.0008502.s002.pdf]

```
JSim v1.1
import nsrunit;
unit conversion on;

/*copyright - Cell Works Group, Inc., 2009 Any reference to this model or derivative
work should contain the following reference."copyright -  Cell Works Group, Inc.,
(2009). Reprinted from www.cellworksgroup.com" */

math ER_PR_PRA_NFKB {
        realDomain t sec;
        t.min = 0;
        t.max = 10000;
        t.delta = 10;
        real PRA_PRB_Ratio(t) dimensionless;
        real EC.Volume L;
        real EC.Vec L;
        real EC.PG.Amount(t) umol;
        real EC.PG.Concentration(t) uM;
        real EC.SHBG_ES.Amount(t) umol;
        real EC.SHBG_ES.Concentration(t) uM;
        real EC.SHBG_ES.InitialConcentration uM;
        real EC.ES.Amount(t) umol;
        real EC.ES.Concentration(t) uM;
        real EC.SHBG_PG.Amount(t) umol;
        real EC.SHBG_PG.Concentration(t) uM;
        real EC.SHBG_PG.InitialConcentration uM;
        real EC.Cyt.Volume L;
        real EC.Cyt.Vcyt L;
        real EC.Cyt.ESRc_mRNA.Amount(t) umol;
        real EC.Cyt.ESRc_mRNA.Concentration(t) uM;
        real EC.Cyt.ESRc_mRNA.InitialConcentration uM;
        real EC.Cyt.VQDPIGKQWC.Amount(t) umol;
        real EC.Cyt.VQDPIGKQWC.Concentration(t) uM;
        real EC.Cyt.VQDPIGKQWC.InitialConcentration uM;
        real EC.Cyt.BDCIKSYKJY.Amount(t) umol;
        real EC.Cyt.BDCIKSYKJY.Concentration(t) uM;
        real EC.Cyt.BDCIKSYKJY.InitialConcentration uM;
        real EC.Cyt.NFKBIAc.Amount(t) umol;
        real EC.Cyt.NFKBIAc.Concentration(t) uM;
        real EC.Cyt.NFKBIAc.InitialConcentration uM;
        real EC.Cyt.TJMSEORQAV.Amount(t) umol;
        real EC.Cyt.TJMSEORQAV.Concentration(t) uM;
        real EC.Cyt.TJMSEORQAV.InitialConcentration uM;
        real EC.Cyt.WSQTUBSXTX.Amount(t) umol;
        real EC.Cyt.WSQTUBSXTX.Concentration(t) uM;
        real EC.Cyt.TBJMXSSCJU.Amount(t) umol;
        real EC.Cyt.TBJMXSSCJU.Concentration(t) uM;
        real EC.Cyt.TBJMXSSCJU.InitialConcentration uM;
        real EC.Cyt.YINGPPRWIO.Amount(t) umol;
        real EC.Cyt.YINGPPRWIO.Concentration(t) uM;
        real EC.Cyt.YINGPPRWIO.InitialConcentration uM;
        real EC.Cyt.VUVKKIUMSM.Amount(t) umol;
        real EC.Cyt.VUVKKIUMSM.Concentration(t) uM;
        real EC.Cyt.VUVKKIUMSM.InitialConcentration uM;
        real EC.Cyt.RFFXXGONQA.Amount(t) umol;
        real EC.Cyt.RFFXXGONQA.Concentration(t) uM;
        real EC.Cyt.RFFXXGONQA.InitialConcentration uM;
        real EC.Cyt.Pr.Volume L;
        real EC.Cyt.Pr.Vpr L;
        EC.Cyt.Pr.Vpr = 0.0166;
        EC.Cyt.Pr.Volume = 0.0166;
        real EC.Cyt.FCMRIEXQWG.Amount(t) umol;
        real EC.Cyt.FCMRIEXQWG.Concentration(t) uM;
        real EC.Cyt.FCMRIEXQWG.InitialConcentration uM;
```

```
real EC.Cyt.ER.Volume L;
real EC.Cyt.ER.Ver L;
EC.Cyt.ER.Ver = 0.1494;
EC.Cyt.ER.Volume = 0.1494;
real EC.Cyt.CHUK_p.Amount(t) umol;
real EC.Cyt.CHUK_p.Concentration(t) uM;
real EC.Cyt.CHUK_p.InitialConcentration uM;
real EC.Cyt.GA.Volume L;
real EC.Cyt.GA.Vga L;
EC.Cyt.GA.Vga = 0.0996;
EC.Cyt.GA.Volume = 0.0996;
real EC.Cyt.ADP.Amount(t) umol;
real EC.Cyt.ADP.Concentration(t) uM;
real EC.Cyt.ADP.InitialConcentration uM;
real EC.Cyt.SMMYREUOEE.Amount(t) umol;
real EC.Cyt.SMMYREUOEE.Concentration(t) uM;
real EC.Cyt.SMMYREUOEE.InitialConcentration uM;
real EC.Cyt.NFKB1c.Amount(t) umol;
real EC.Cyt.NFKB1c.Concentration(t) uM;
real EC.Cyt.NFKB1c.InitialConcentration uM;
real EC.Cyt.MEUHHCXDNY.Amount(t) umol;
real EC.Cyt.MEUHHCXDNY.Concentration(t) uM;
real EC.Cyt.MEUHHCXDNY.InitialConcentration uM;
real EC.Cyt.PGRB.Amount(t) umol;
real EC.Cyt.PGRB.Concentration(t) uM;
real EC.Cyt.PGRB.InitialConcentration uM;
real EC.Cyt.XKVYWVGFII.Amount(t) umol;
real EC.Cyt.XKVYWVGFII.Concentration(t) uM;
real EC.Cyt.XKVYWVGFII.InitialConcentration uM;
real EC.Cyt.KMXEIYOWMQ.Amount(t) umol;
real EC.Cyt.KMXEIYOWMQ.Concentration(t) uM;
real EC.Cyt.KMXEIYOWMQ.InitialConcentration uM;
real EC.Cyt.IVPLRKCCWI.Amount(t) umol;
real EC.Cyt.IVPLRKCCWI.Concentration(t) uM;
real EC.Cyt.IVPLRKCCWI.InitialConcentration uM;
real EC.Cyt.LBXTYHSEFK.Amount(t) umol;
real EC.Cyt.LBXTYHSEFK.Concentration(t) uM;
real EC.Cyt.LBXTYHSEFK.InitialConcentration uM;
real EC.Cyt.SFKBLJATXG.Amount(t) umol;
real EC.Cyt.SFKBLJATXG.Concentration(t) uM;
real EC.Cyt.SFKBLJATXG.InitialConcentration uM;
real EC.Cyt.PGRBc_mRNA.Amount(t) umol;
real EC.Cyt.PGRBc_mRNA.Concentration(t) uM;
real EC.Cyt.PGRBc_mRNA.InitialConcentration uM;
real EC.Cyt.PGRA.Amount(t) umol;
real EC.Cyt.PGRA.Concentration(t) uM;
real EC.Cyt.PGRA.InitialConcentration uM;
real EC.Cyt.VXLSQVSTNA.Amount(t) umol;
real EC.Cyt.VXLSQVSTNA.Concentration(t) uM;
real EC.Cyt.VXLSQVSTNA.InitialConcentration uM;
real EC.Cyt.Ly.Volume L;
real EC.Cyt.Ly.Vly L;
EC.Cyt.Ly.Vly = 0.0166;
EC.Cyt.Ly.Volume = 0.0166;
real EC.Cyt.HSP90AA1_pool.Amount(t) umol;
real EC.Cyt.HSP90AA1_pool.Concentration(t) uM;
real EC.Cyt.HSP90AA1_pool.InitialConcentration uM;
real EC.Cyt.EFUYJJRUFC.Amount(t) umol;
real EC.Cyt.EFUYJJRUFC.Concentration(t) uM;
real EC.Cyt.EFUYJJRUFC.InitialConcentration uM;
real EC.Cyt.BUKQBUVSPL.Amount(t) umol;
real EC.Cyt.BUKQBUVSPL.Concentration(t) uM;
real EC.Cyt.BUKQBUVSPL.InitialConcentration uM;
real EC.Cyt.IBUAUBLBAJ.Amount(t) umol;
```

```
real EC.Cyt.IBUAUBLBAJ.Concentration(t) uM;
real EC.Cyt.IBUAUBLBAJ.InitialConcentration uM;
real EC.Cyt.QQDSAXKYSH.Amount(t) umol;
real EC.Cyt.QQDSAXKYSH.Concentration(t) uM;
real EC.Cyt.QQDSAXKYSH.InitialConcentration uM;
real EC.Cyt.ATP.Amount(t) umol;
real EC.Cyt.ATP.Concentration(t) uM;
real EC.Cyt.ATP.InitialConcentration uM;
real EC.Cyt.JWYDCVVNFY.Amount(t) umol;
real EC.Cyt.JWYDCVVNFY.Concentration(t) uM;
real EC.Cyt.JWYDCVVNFY.InitialConcentration uM;
real EC.Cyt.ESR1.Amount(t) umol;
real EC.Cyt.ESR1.Concentration(t) uM;
real EC.Cyt.ESR1.InitialConcentration uM;
real EC.Cyt.IHYPKQPWTX.Amount(t) umol;
real EC.Cyt.IHYPKQPWTX.Concentration(t) uM;
real EC.Cyt.IHYPKQPWTX.InitialConcentration uM;
real EC.Cyt.GVEAOFSCUE.Amount(t) umol;
real EC.Cyt.GVEAOFSCUE.Concentration(t) uM;
real EC.Cyt.GVEAOFSCUE.InitialConcentration uM;
real EC.Cyt.PGRAc_mRNA.Amount(t) umol;
real EC.Cyt.PGRAc_mRNA.Concentration(t) uM;
real EC.Cyt.PGRAc_mRNA.InitialConcentration uM;
real EC.Cyt.VESHECAPCO.Amount(t) umol;
real EC.Cyt.VESHECAPCO.Concentration(t) uM;
real EC.Cyt.VESHECAPCO.InitialConcentration uM;
real EC.Cyt.CUUPSCJFCK.Amount(t) umol;
real EC.Cyt.CUUPSCJFCK.Concentration(t) uM;
real EC.Cyt.CUUPSCJFCK.InitialConcentration uM;
real EC.Cyt.IHRRYRSKWQ.Amount(t) umol;
real EC.Cyt.IHRRYRSKWQ.Concentration(t) uM;
real EC.Cyt.IHRRYRSKWQ.InitialConcentration uM;
real EC.Cyt.CO.Amount(t) umol;
real EC.Cyt.CO.Concentration(t) uM;
real EC.Cyt.CO.InitialConcentration uM;
real EC.Cyt.VCKNCEABFO.Amount(t) umol;
real EC.Cyt.VCKNCEABFO.Concentration(t) uM;
real EC.Cyt.VCKNCEABFO.InitialConcentration uM;
real EC.Cyt.Mt.Volume L;
real EC.Cyt.Mt.Vmt L;
EC.Cyt.Mt.Vmt = 0.3652;
EC.Cyt.Mt.Volume = 0.3652;
real EC.Cyt.HXPQSSVMJO.Amount(t) umol;
real EC.Cyt.HXPQSSVMJO.Concentration(t) uM;
real EC.Cyt.HXPQSSVMJO.InitialConcentration uM;
real EC.Cyt.IIYBUWADRK.Amount(t) umol;
real EC.Cyt.IIYBUWADRK.Concentration(t) uM;
real EC.Cyt.IIYBUWADRK.InitialConcentration uM;
real EC.Cyt.N.Volume L;
real EC.Cyt.N.Vn L;
real EC.Cyt.N.FAAYBECMNJ.Amount(t) umol;
real EC.Cyt.N.FAAYBECMNJ.Concentration(t) uM;
real EC.Cyt.N.FAAYBECMNJ.InitialConcentration uM;
real EC.Cyt.N.PIBFOAUQHQ.Amount(t) umol;
real EC.Cyt.N.PIBFOAUQHQ.Concentration(t) uM;
real EC.Cyt.N.PIBFOAUQHQ.InitialConcentration uM;
real EC.Cyt.N.MTACYBTIEX.Amount(t) umol;
real EC.Cyt.N.MTACYBTIEX.Concentration(t) uM;
real EC.Cyt.N.MTACYBTIEX.InitialConcentration uM;
real EC.Cyt.N.PWJCQEHQNG.Amount(t) umol;
real EC.Cyt.N.PWJCQEHQNG.Concentration(t) uM;
real EC.Cyt.N.PWJCQEHQNG.InitialConcentration uM;
real EC.Cyt.N.DONQANHYGT.Amount(t) umol;
real EC.Cyt.N.DONQANHYGT.Concentration(t) uM;
```

```
real EC.Cyt.N.DONQANHYGT.InitialConcentration uM;
real EC.Cyt.N.LSPVTOVYYU.Amount(t) umol;
real EC.Cyt.N.LSPVTOVYYU.Concentration(t) uM;
real EC.Cyt.N.LSPVTOVYYU.InitialConcentration uM;
real EC.Cyt.N.MDTAOIEDKY.Amount(t) umol;
real EC.Cyt.N.MDTAOIEDKY.Concentration(t) uM;
real EC.Cyt.N.MDTAOIEDKY.InitialConcentration uM;
real EC.Cyt.N.NOCWORPSMX.Amount(t) umol;
real EC.Cyt.N.NOCWORPSMX.Concentration(t) uM;
real EC.Cyt.N.NOCWORPSMX.InitialConcentration uM;
real EC.Cyt.N.QKSUBHODFF.Amount(t) umol;
real EC.Cyt.N.QKSUBHODFF.Concentration(t) uM;
real EC.Cyt.N.QKSUBHODFF.InitialConcentration uM;
real EC.Cyt.N.HIIDJNHNHE.Amount(t) umol;
real EC.Cyt.N.HIIDJNHNHE.Concentration(t) uM;
real EC.Cyt.N.HIIDJNHNHE.InitialConcentration uM;
real EC.Cyt.N.CAAYFXCTLG.Amount(t) umol;
real EC.Cyt.N.CAAYFXCTLG.Concentration(t) uM;
real EC.Cyt.N.CAAYFXCTLG.InitialConcentration uM;
real EC.Cyt.N.VWLRCPRFDD.Amount(t) umol;
real EC.Cyt.N.VWLRCPRFDD.Concentration(t) uM;
real EC.Cyt.N.VWLRCPRFDD.InitialConcentration uM;
real EC.Cyt.N.NFKB1n.Amount(t) umol;
real EC.Cyt.N.NFKB1n.Concentration(t) uM;
real EC.Cyt.N.NFKB1n.InitialConcentration uM;
real EC.Cyt.N.XXMIPNQUOL.Amount(t) umol;
real EC.Cyt.N.XXMIPNQUOL.Concentration(t) uM;
real EC.Cyt.N.XXMIPNQUOL.InitialConcentration uM;
real EC.Cyt.N.MKNUYLQQGK.Amount(t) umol;
real EC.Cyt.N.MKNUYLQQGK.Concentration(t) uM;
real EC.Cyt.N.MKNUYLQQGK.InitialConcentration uM;
real EC.Cyt.N.NKGFVKNAGT.Amount(t) umol;
real EC.Cyt.N.NKGFVKNAGT.Concentration(t) uM;
real EC.Cyt.N.NKGFVKNAGT.InitialConcentration uM;
real EC.Cyt.N.IFRVPCCTBU.Amount(t) umol;
real EC.Cyt.N.IFRVPCCTBU.Concentration(t) uM;
real EC.Cyt.N.IFRVPCCTBU.InitialConcentration uM;
real EC.Cyt.N.AJIYNVOQJR.Amount(t) umol;
real EC.Cyt.N.AJIYNVOQJR.Concentration(t) uM;
real EC.Cyt.N.AJIYNVOQJR.InitialConcentration uM;
real EC.Cyt.N.NRKRVULXIY.Amount(t) umol;
real EC.Cyt.N.NRKRVULXIY.Concentration(t) uM;
real EC.Cyt.N.NRKRVULXIY.InitialConcentration uM;
real EC.Cyt.N.DWPMBRWHEF.Amount(t) umol;
real EC.Cyt.N.DWPMBRWHEF.Concentration(t) uM;
real EC.Cyt.N.DWPMBRWHEF.InitialConcentration uM;
real EC.Cyt.N.VBOAMHRWEU.Amount(t) umol;
real EC.Cyt.N.VBOAMHRWEU.Concentration(t) uM;
real EC.Cyt.N.VBOAMHRWEU.InitialConcentration uM;
real EC.Cyt.N.XUKHUJBSKY.Amount(t) umol;
real EC.Cyt.N.XUKHUJBSKY.Concentration(t) uM;
real EC.Cyt.N.XUKHUJBSKY.InitialConcentration uM;
real EC.Cyt.N.LEADDBWRAG.Amount(t) umol;
real EC.Cyt.N.LEADDBWRAG.Concentration(t) uM;
real EC.Cyt.N.LEADDBWRAG.InitialConcentration uM;
real EC.Cyt.N.AGOBMLVSUW.Amount(t) umol;
real EC.Cyt.N.AGOBMLVSUW.Concentration(t) uM;
real EC.Cyt.N.AGOBMLVSUW.InitialConcentration uM;
real EC.Cyt.N.CVVQVNKOYS.Amount(t) umol;
real EC.Cyt.N.CVVQVNKOYS.Concentration(t) uM;
real EC.Cyt.N.CVVQVNKOYS.InitialConcentration uM;
real EC.Cyt.N.WXMNCUYFYD.Amount(t) umol;
real EC.Cyt.N.WXMNCUYFYD.Concentration(t) uM;
real EC.Cyt.N.WXMNCUYFYD.InitialConcentration uM;
```

```
        real EC.Cyt.N.JKLDQAETKY.Amount(t) umol;
        real EC.Cyt.N.JKLDQAETKY.Concentration(t) uM;
        real EC.Cyt.N.JKLDQAETKY.InitialConcentration uM;
        real EC.Cyt.N.QBUVTSDESA.Amount(t) umol;
        real EC.Cyt.N.QBUVTSDESA.Concentration(t) uM;
        real EC.Cyt.N.QBUVTSDESA.InitialConcentration uM;
        real EC.Cyt.N.KYYFWUUFJR.Amount(t) umol;
        real EC.Cyt.N.KYYFWUUFJR.Concentration(t) uM;
        real EC.Cyt.N.KYYFWUUFJR.InitialConcentration uM;
        real EC.Cyt.N.LXLRJTCMCN.Amount(t) umol;
        real EC.Cyt.N.LXLRJTCMCN.Concentration(t) uM;
        real EC.Cyt.N.LXLRJTCMCN.InitialConcentration uM;
        real EC.Cyt.N.LEPXNCOIXH.Amount(t) umol;
        real EC.Cyt.N.LEPXNCOIXH.Concentration(t) uM;
        real EC.Cyt.N.LEPXNCOIXH.InitialConcentration uM;
        real EC.Cyt.N.LIKUEPJGFI.Amount(t) umol;
        real EC.Cyt.N.LIKUEPJGFI.Concentration(t) uM;
        real EC.Cyt.N.LIKUEPJGFI.InitialConcentration uM;
        real EC.Cyt.N.VATMHXVNSV.Amount(t) umol;
        real EC.Cyt.N.VATMHXVNSV.Concentration(t) uM;
        real EC.Cyt.N.VATMHXVNSV.InitialConcentration uM;
        real EC.Cyt.N.DWWTIFITMT.Amount(t) umol;
        real EC.Cyt.N.DWWTIFITMT.Concentration(t) uM;
        real EC.Cyt.N.DWWTIFITMT.InitialConcentration uM;
        real EC.Cyt.N.TLGVYSXTLN.Amount(t) umol;
        real EC.Cyt.N.TLGVYSXTLN.Concentration(t) uM;
        real EC.Cyt.N.TLGVYSXTLN.InitialConcentration uM;
        real EC.Cyt.N.ES_ESR1_Di.Flux(t) umol/sec;
        real EC.Cyt.N.ES_ESR1_Di.Kf 1/(uM*sec);
        EC.Cyt.N.ES_ESR1_Di.Kf = 23/60;
        EC.Cyt.N.ES_ESR1_Di.Flux = (EC.Cyt.N.ES_ESR1_Di.Kf*
(EC.Cyt.N.LXLRJTCMCN.Concentration^2))*EC.Cyt.N.Vn;
        real EC.Cyt.N.PGRA_NCOA_AC.Flux(t) umol/sec;
        real EC.Cyt.N.PGRA_NCOA_AC.Kf 1/(uM*sec);
        real EC.Cyt.N.PGRA_NCOA_AC.Kr 1/sec;
        EC.Cyt.N.PGRA_NCOA_AC.Kf = 0.3;
        EC.Cyt.N.PGRA_NCOA_AC.Flux =
(((EC.Cyt.N.PGRA_NCOA_AC.Kf*EC.Cyt.N.QKSUBHODFF.Concentration)
*EC.Cyt.N.AGOBMLVSUW.Concentration)-
(EC.Cyt.N.PGRA_NCOA_AC.Kr*EC.Cyt.N.NKGFVKNAGT.Concentration))*EC.Cyt.N.Vn;
        EC.Cyt.N.PGRA_NCOA_AC.Kr = 0.0040;
        real EC.Cyt.N.ESRmRNA_Txn.Flux(t) umol/sec;
        real EC.Cyt.N.ESRmRNA_Txn.Vf umol/sec;
        real EC.Cyt.N.ESRmRNA_Txn.Ki uM;
        real EC.Cyt.N.ESRmRNA_Txn.Km_ESR uM;
        EC.Cyt.N.ESRmRNA_Txn.Vf = 0.01;
        EC.Cyt.N.ESRmRNA_Txn.Ki = 0.0050;
        EC.Cyt.N.ESRmRNA_Txn.Flux =
(EC.Cyt.N.ESRmRNA_Txn.Vf*EC.Cyt.N.MTACYBTIEX.Concentration)/
((EC.Cyt.N.ESRmRNA_Txn.Km_ESR*(1+(EC.Cyt.N.AJIYNVOQJR.Concentration/
EC.Cyt.N.ESRmRNA_Txn.Ki)))+EC.Cyt.N.MTACYBTIEX.Concentration);
        EC.Cyt.N.ESRmRNA_Txn.Km_ESR = 3;
        real EC.Cyt.N.PG_PGRA_Di.Flux(t) umol/sec;
        real EC.Cyt.N.PG_PGRA_Di.Kf 1/(uM*sec);
        real EC.Cyt.N.PG_PGRA_Di.Kr 1/sec;
        EC.Cyt.N.PG_PGRA_Di.Kf = 1;
        EC.Cyt.N.PG_PGRA_Di.Flux = ((EC.Cyt.N.PG_PGRA_Di.Kf*
(EC.Cyt.N.CAAYFXCTLG.Concentration^2))-
(EC.Cyt.N.PG_PGRA_Di.Kr*EC.Cyt.N.CVVQVNKOYS.Concentration))*EC.Cyt.N.Vn;
        EC.Cyt.N.PG_PGRA_Di.Kr = 0.0070;
        real EC.Cyt.N.PGRmRNAn_Dgr.Flux(t) umol/sec;
        real EC.Cyt.N.PGRmRNAn_Dgr.Kf 1/sec;
        EC.Cyt.N.PGRmRNAn_Dgr.Kf = 1.0E-4;
        EC.Cyt.N.PGRmRNAn_Dgr.Flux =
```

```
(EC.Cyt.N.PGRmRNAn_Dgr.Kf*EC.Cyt.N.LSPVTOVYYU.Concentration)*EC.Cyt.N.Vn;
        real EC.Cyt.N.TC2_CBP_Bd.Flux(t) umol/sec;
        real EC.Cyt.N.TC2_CBP_Bd.Kf 1/(uM*sec);
        real EC.Cyt.N.TC2_CBP_Bd.Kr 1/sec;
        EC.Cyt.N.TC2_CBP_Bd.Kf = 1;
        EC.Cyt.N.TC2_CBP_Bd.Flux =
(((EC.Cyt.N.TC2_CBP_Bd.Kf*EC.Cyt.N.XUKHUJBSKY.Concentration)
*EC.Cyt.N.DONQANHYGT.Concentration)-
(EC.Cyt.N.TC2_CBP_Bd.Kr*EC.Cyt.N.LIKUEPJGFI.Concentration))*EC.Cyt.N.Vn;
        EC.Cyt.N.TC2_CBP_Bd.Kr = 1.0E-4;
        real EC.Cyt.N.TC1_NCOA_AC.Flux(t) umol/sec;
        real EC.Cyt.N.TC1_NCOA_AC.Kf 1/(uM*sec);
        real EC.Cyt.N.TC1_NCOA_AC.Kr 1/sec;
        EC.Cyt.N.TC1_NCOA_AC.Kf = 0.45;
        EC.Cyt.N.TC1_NCOA_AC.Flux =
(((EC.Cyt.N.TC1_NCOA_AC.Kf*EC.Cyt.N.PWJCQEHQNG.Concentration)
*EC.Cyt.N.QKSUBHODFF.Concentration)-
(EC.Cyt.N.TC1_NCOA_AC.Kr*EC.Cyt.N.DONQANHYGT.Concentration))*EC.Cyt.N.Vn;
        EC.Cyt.N.TC1_NCOA_AC.Kr = 0.0040;
        real EC.Cyt.N.TPA2_CBP_Bd.Flux(t) umol/sec;
        real EC.Cyt.N.TPA2_CBP_Bd.Kf 1/(uM*sec);
        real EC.Cyt.N.TPA2_CBP_Bd.Kr 1/sec;
        EC.Cyt.N.TPA2_CBP_Bd.Kf = 0.1;
        EC.Cyt.N.TPA2_CBP_Bd.Flux =
(((EC.Cyt.N.TPA2_CBP_Bd.Kf*EC.Cyt.N.NKGFVKNAGT.Concentration)
*EC.Cyt.N.XUKHUJBSKY.Concentration)-
(EC.Cyt.N.TPA2_CBP_Bd.Kr*EC.Cyt.N.NRKRVULXIY.Concentration))*EC.Cyt.N.Vn;
        EC.Cyt.N.TPA2_CBP_Bd.Kr = 1.0E-4;
        real EC.Cyt.N.TP3_PCAF_Bd.Flux(t) umol/sec;
        real EC.Cyt.N.TP3_PCAF_Bd.Kf 1/(uM*sec);
        real EC.Cyt.N.TP3_PCAF_Bd.Ki uM;
        real EC.Cyt.N.TP3_PCAF_Bd.Kr 1/sec;
        EC.Cyt.N.TP3_PCAF_Bd.Kf = 2;
        EC.Cyt.N.TP3_PCAF_Bd.Ki = 6.0E-5;
        EC.Cyt.N.TP3_PCAF_Bd.Flux = ((((EC.Cyt.N.TP3_PCAF_Bd.Kf/(1
+(EC.Cyt.N.NFKB1n.Concentration/EC.Cyt.N.TP3_PCAF_Bd.Ki)))
*EC.Cyt.N.IFRVPCCTBU.Concentration)*EC.Cyt.N.VATMHXVNSV.Concentration)-
(EC.Cyt.N.TP3_PCAF_Bd.Kr*EC.Cyt.N.FAAYBECMNJ.Concentration))*EC.Cyt.N.Vn;
        EC.Cyt.N.TP3_PCAF_Bd.Kr = 0.01;
        real EC.Cyt.N.NFKBIAn_NFKB1n_Tr.Kf umol/(uM*sec);
        real EC.Cyt.N.NFKBIAn_NFKB1n_Tr.Flux(t) umol/sec;
        real EC.Cyt.N.NFKBIAn_NFKB1n_Tr.Kr umol/(uM*sec);
        EC.Cyt.N.NFKBIAn_NFKB1n_Tr.Flux =
(EC.Cyt.N.NFKBIAn_NFKB1n_Tr.Kf*EC.Cyt.N.LEADDBWRAG.Concentration)-
(EC.Cyt.N.NFKBIAn_NFKB1n_Tr.Kr*EC.Cyt.MEUHHCXDNY.Concentration);
        EC.Cyt.N.NFKBIAn_NFKB1n_Tr.Kf = 0.8294;
        EC.Cyt.N.NFKBIAn_NFKB1n_Tr.Kr = 0;
        real EC.Cyt.N.TC4_TRAP_Bd.Flux(t) umol/sec;
        real EC.Cyt.N.TC4_TRAP_Bd.Kf 1/(uM*sec);
        real EC.Cyt.N.TC4_TRAP_Bd.Kr 1/sec;
        EC.Cyt.N.TC4_TRAP_Bd.Kf = 0.01;
        EC.Cyt.N.TC4_TRAP_Bd.Flux =
(((EC.Cyt.N.TC4_TRAP_Bd.Kf*EC.Cyt.N.JKLDQAETKY.Concentration)
*EC.Cyt.N.DWPMBRWHEF.Concentration)-
(EC.Cyt.N.TC4_TRAP_Bd.Kr*EC.Cyt.N.MTACYBTIEX.Concentration))*EC.Cyt.N.Vn;
        EC.Cyt.N.TC4_TRAP_Bd.Kr = 0.0086;
        real EC.Cyt.N.PGRB_PRE_Bd.Flux(t) umol/sec;
        real EC.Cyt.N.PGRB_PRE_Bd.Kf 1/(uM*sec);
        real EC.Cyt.N.PGRB_PRE_Bd.Kr 1/sec;
        EC.Cyt.N.PGRB_PRE_Bd.Kf = 0.1;
        EC.Cyt.N.PGRB_PRE_Bd.Flux =
(((EC.Cyt.N.PGRB_PRE_Bd.Kf*EC.Cyt.N.LEPXNCOIXH.Concentration)
*EC.Cyt.N.WXMNCUYFYD.Concentration)-
(EC.Cyt.N.PGRB_PRE_Bd.Kr*EC.Cyt.N.VBOAMHRWEU.Concentration))*EC.Cyt.N.Vn;
```

```
        EC.Cyt.N.PGRB_PRE_Bd.Kr = 0.0013;
        real EC.Cyt.N.TP2_CBP_Bd.Flux(t) umol/sec;
        real EC.Cyt.N.TP2_CBP_Bd.Kf 1/(uM*sec);
        real EC.Cyt.N.TP2_CBP_Bd.Kr 1/sec;
        EC.Cyt.N.TP2_CBP_Bd.Kf = 10;
        EC.Cyt.N.TP2_CBP_Bd.Flux =
(((EC.Cyt.N.TP2_CBP_Bd.Kf*EC.Cyt.N.DWWTIFITMT.Concentration)
*EC.Cyt.N.XUKHUJBSKY.Concentration)-
(EC.Cyt.N.TP2_CBP_Bd.Kr*EC.Cyt.N.IFRVPCCTBU.Concentration))*EC.Cyt.N.Vn;
        EC.Cyt.N.TP2_CBP_Bd.Kr = 1.0E-4;
        real EC.Cyt.N.PGRAmRNAn_Dgr.Flux(t) umol/sec;
        real EC.Cyt.N.PGRAmRNAn_Dgr.Kf 1/sec;
        EC.Cyt.N.PGRAmRNAn_Dgr.Kf = 1.0E-4;
        EC.Cyt.N.PGRAmRNAn_Dgr.Flux =
(EC.Cyt.N.PGRAmRNAn_Dgr.Kf*EC.Cyt.N.KYYFWUUFJR.Concentration)*EC.Cyt.N.Vn;
        real EC.Cyt.N.TP4_TRAP_Bd.Flux(t) umol/sec;
        real EC.Cyt.N.TP4_TRAP_Bd.Kf 1/(uM*sec);
        real EC.Cyt.N.TP4_TRAP_Bd.Kr 1/sec;
        EC.Cyt.N.TP4_TRAP_Bd.Kf = 0.01;
        EC.Cyt.N.TP4_TRAP_Bd.Flux =
(((EC.Cyt.N.TP4_TRAP_Bd.Kf*EC.Cyt.N.FAAYBECMNJ.Concentration)
*EC.Cyt.N.JKLDQAETKY.Concentration)-
(EC.Cyt.N.TP4_TRAP_Bd.Kr*EC.Cyt.N.AJIYNVOQJR.Concentration))*EC.Cyt.N.Vn;
        EC.Cyt.N.TP4_TRAP_Bd.Kr = 0.0086;
        real EC.Cyt.N.ESRmRNAn_Dgr.Flux(t) umol/sec;
        real EC.Cyt.N.ESRmRNAn_Dgr.Kf 1/sec;
        EC.Cyt.N.ESRmRNAn_Dgr.Kf = 1.0E-4;
        EC.Cyt.N.ESRmRNAn_Dgr.Flux =
(EC.Cyt.N.ESRmRNAn_Dgr.Kf*EC.Cyt.N.MKNUYLQQGK.Concentration)*EC.Cyt.N.Vn;
        real EC.Cyt.N.PGRBmRNA_Txn.Flux(t) umol/sec;
        real EC.Cyt.N.PGRBmRNA_Txn.Km_PGRB uM;
        real EC.Cyt.N.PGRBmRNA_Txn.Vf umol/sec;
        real EC.Cyt.N.PGRBmRNA_Txn.Ki uM;
        EC.Cyt.N.PGRBmRNA_Txn.Km_PGRB = 1;
        EC.Cyt.N.PGRBmRNA_Txn.Vf = 0.05;
        EC.Cyt.N.PGRBmRNA_Txn.Ki = 0.01;
        EC.Cyt.N.PGRBmRNA_Txn.Flux =
(EC.Cyt.N.PGRBmRNA_Txn.Vf*EC.Cyt.N.AJIYNVOQJR.Concentration)/
((EC.Cyt.N.PGRBmRNA_Txn.Km_PGRB*(1+(EC.Cyt.N.NFKB1n.Concentration/
EC.Cyt.N.PGRBmRNA_Txn.Ki)))+EC.Cyt.N.AJIYNVOQJR.Concentration);
        real EC.Cyt.N.PGRB_NCOA_AC.Flux(t) umol/sec;
        real EC.Cyt.N.PGRB_NCOA_AC.Kf 1/(uM*sec);
        real EC.Cyt.N.PGRB_NCOA_AC.Kr 1/sec;
        EC.Cyt.N.PGRB_NCOA_AC.Kf = 0.5;
        EC.Cyt.N.PGRB_NCOA_AC.Flux =
(((EC.Cyt.N.PGRB_NCOA_AC.Kf*EC.Cyt.N.VBOAMHRWEU.Concentration)
*EC.Cyt.N.QKSUBHODFF.Concentration)-
(EC.Cyt.N.PGRB_NCOA_AC.Kr*EC.Cyt.N.DWWTIFITMT.Concentration))*EC.Cyt.N.Vn;
        EC.Cyt.N.PGRB_NCOA_AC.Kr = 0.0040;
        real EC.Cyt.N.NFKBIAn_NFKB1n_Bd.Flux(t) umol/sec;
        real EC.Cyt.N.NFKBIAn_NFKB1n_Bd.Kf 1/(uM*sec);
        real EC.Cyt.N.NFKBIAn_NFKB1n_Bd.Kr 1/sec;
        EC.Cyt.N.NFKBIAn_NFKB1n_Bd.Kf = 0.5;
        EC.Cyt.N.NFKBIAn_NFKB1n_Bd.Flux =
(((EC.Cyt.N.NFKBIAn_NFKB1n_Bd.Kf*EC.Cyt.N.NFKB1n.Concentration)
*EC.Cyt.N.VWLRCPRFDD.Concentration)-
(EC.Cyt.N.NFKBIAn_NFKB1n_Bd.Kr*EC.Cyt.N.LEADDBWRAG.Concentration))*EC.Cyt.N.Vn;
        EC.Cyt.N.NFKBIAn_NFKB1n_Bd.Kr = 5.0E-4;
        real EC.Cyt.N.PGRAmRNA_Txn.Flux(t) umol/sec;
        real EC.Cyt.N.PGRAmRNA_Txn.Vf umol/sec;
        real EC.Cyt.N.PGRAmRNA_Txn.Ki uM;
        real EC.Cyt.N.PGRAmRNA_Txn.Km_PGRA uM;
        EC.Cyt.N.PGRAmRNA_Txn.Vf = 0.01;
        EC.Cyt.N.PGRAmRNA_Txn.Ki = 0.0050;
```

```
        EC.Cyt.N.PGRAmRNA_Txn.Km_PGRA = 4;
        EC.Cyt.N.PGRAmRNA_Txn.Flux =
(EC.Cyt.N.PGRAmRNA_Txn.Vf*EC.Cyt.N.MTACYBTIEX.Concentration)/
((EC.Cyt.N.PGRAmRNA_Txn.Km_PGRA*(1+(EC.Cyt.N.AJIYNVOQJR.Concentration/
EC.Cyt.N.PGRAmRNA_Txn.Ki)))+EC.Cyt.N.MTACYBTIEX.Concentration);
        real EC.Cyt.N.TC3_BRG_Bd.Flux(t) umol/sec;
        real EC.Cyt.N.TC3_BRG_Bd.Kf 1/(uM*sec);
        real EC.Cyt.N.TC3_BRG_Bd.Ki uM;
        real EC.Cyt.N.TC3_BRG_Bd.Kr 1/sec;
        EC.Cyt.N.TC3_BRG_Bd.Kf = 1;
        EC.Cyt.N.TC3_BRG_Bd.Ki = 1.0E-5;
        EC.Cyt.N.TC3_BRG_Bd.Flux = ((((EC.Cyt.N.TC3_BRG_Bd.Kf/(1
+(EC.Cyt.N.FAAYBECMNJ.Concentration/EC.Cyt.N.TC3_BRG_Bd.Ki)))
*EC.Cyt.N.LIKUEPJGFI.Concentration)*EC.Cyt.N.TLGVYSXTLN.Concentration)-
(EC.Cyt.N.TC3_BRG_Bd.Kr*EC.Cyt.N.DWPMBRWHEF.Concentration))*EC.Cyt.N.Vn;
        EC.Cyt.N.TC3_BRG_Bd.Kr = 0.01;
        real EC.Cyt.N.PG_PGRB_Di.Flux(t) umol/sec;
        real EC.Cyt.N.PG_PGRB_Di.Kf 1/(uM*sec);
        real EC.Cyt.N.PG_PGRB_Di.Kr 1/sec;
        EC.Cyt.N.PG_PGRB_Di.Kf = 1;
        EC.Cyt.N.PG_PGRB_Di.Flux = ((EC.Cyt.N.PG_PGRB_Di.Kf*
(EC.Cyt.N.XXMIPNQUOL.Concentration^2))-
(EC.Cyt.N.PG_PGRB_Di.Kr*EC.Cyt.N.WXMNCUYFYD.Concentration))*EC.Cyt.N.Vn;
        EC.Cyt.N.PG_PGRB_Di.Kr = 0.0070;
        real EC.Cyt.N.TPA3_PCAF_Bd.Flux(t) umol/sec;
        real EC.Cyt.N.TPA3_PCAF_Bd.Kf 1/(uM*sec);
        real EC.Cyt.N.TPA3_PCAF_Bd.Kr 1/sec;
        EC.Cyt.N.TPA3_PCAF_Bd.Kf = 1;
        EC.Cyt.N.TPA3_PCAF_Bd.Flux =
(((EC.Cyt.N.TPA3_PCAF_Bd.Kf*EC.Cyt.N.NRKRVULXIY.Concentration)
*EC.Cyt.N.VATMHXVNSV.Concentration)-
(EC.Cyt.N.TPA3_PCAF_Bd.Kr*EC.Cyt.N.MDTAOIEDKY.Concentration))*EC.Cyt.N.Vn;
        EC.Cyt.N.TPA3_PCAF_Bd.Kr = 0.01;
        real EC.Cyt.N.PGRA_PRE_Bd.Flux(t) umol/sec;
        real EC.Cyt.N.PGRA_PRE_Bd.Kf 1/(uM*sec);
        real EC.Cyt.N.PGRA_PRE_Bd.Kr 1/sec;
        EC.Cyt.N.PGRA_PRE_Bd.Kf = 0.1;
        EC.Cyt.N.PGRA_PRE_Bd.Flux =
(((EC.Cyt.N.PGRA_PRE_Bd.Kf*EC.Cyt.N.LEPXNCOIXH.Concentration)
*EC.Cyt.N.CVVQVNKOYS.Concentration)-
(EC.Cyt.N.PGRA_PRE_Bd.Kr*EC.Cyt.N.AGOBMLVSUW.Concentration))*EC.Cyt.N.Vn;
        EC.Cyt.N.PGRA_PRE_Bd.Kr = 0.0013;
        real EC.Cyt.N.TPA4_TRAP_Bd.Flux(t) umol/sec;
        real EC.Cyt.N.TPA4_TRAP_Bd.Kf 1/(uM*sec);
        real EC.Cyt.N.TPA4_TRAP_Bd.Kr 1/sec;
        EC.Cyt.N.TPA4_TRAP_Bd.Kf = 0.01;
        EC.Cyt.N.TPA4_TRAP_Bd.Flux =
(((EC.Cyt.N.TPA4_TRAP_Bd.Kf*EC.Cyt.N.MDTAOIEDKY.Concentration)
*EC.Cyt.N.JKLDQAETKY.Concentration)-
(EC.Cyt.N.TPA4_TRAP_Bd.Kr*EC.Cyt.N.QBUVTSDESA.Concentration))*EC.Cyt.N.Vn;
        EC.Cyt.N.TPA4_TRAP_Bd.Kr = 0.0086;
        real EC.Cyt.N.ESR_ERE_Bd.Flux(t) umol/sec;
        real EC.Cyt.N.ESR_ERE_Bd.Kf 1/(uM*sec);
        real EC.Cyt.N.ESR_ERE_Bd.Kr 1/sec;
        EC.Cyt.N.ESR_ERE_Bd.Kf = 0.0962;
        EC.Cyt.N.ESR_ERE_Bd.Flux =
(((EC.Cyt.N.ESR_ERE_Bd.Kf*EC.Cyt.N.NOCWORPSMX.Concentration)
*EC.Cyt.N.HIIDJNHNHE.Concentration)-
(EC.Cyt.N.ESR_ERE_Bd.Kr*EC.Cyt.N.PWJCQEHQNG.Concentration))*EC.Cyt.N.Vn;
        EC.Cyt.N.ESR_ERE_Bd.Kr = 0.00186;
        EC.Cyt.N.Vn = 0.0996;
        EC.Cyt.N.Volume = 0.0996;
        EC.Cyt.N.FAAYBECMNJ.InitialConcentration = 0;
        EC.Cyt.N.PIBFOAUQHQ.InitialConcentration = 0.008454;
```

```
        EC.Cyt.N.MTACYBTIEX.InitialConcentration = 0;
        EC.Cyt.N.PWJCQEHQNG.InitialConcentration = 0;
        EC.Cyt.N.DONQANHYGT.InitialConcentration = 0;
        EC.Cyt.N.LSPVTOVYYU.InitialConcentration = 1.0E-4;
        EC.Cyt.N.MDTAOIEDKY.InitialConcentration = 0;
        EC.Cyt.N.NOCWORPSMX.InitialConcentration = 0;
        EC.Cyt.N.QKSUBHODFF.InitialConcentration = 1;
        EC.Cyt.N.HIIDJNHNHE.InitialConcentration = 0.1;
        EC.Cyt.N.CAAYFXCTLG.InitialConcentration = 0;
        EC.Cyt.N.VWLRCPRFDD.InitialConcentration = 0;
        EC.Cyt.N.NFKB1n.InitialConcentration = 0;
        EC.Cyt.N.XXMIPNQUOL.InitialConcentration = 0;
        EC.Cyt.N.MKNUYLQQGK.InitialConcentration = 1.0E-4;
        EC.Cyt.N.NKGFVKNAGT.InitialConcentration = 0;
        EC.Cyt.N.IFRVPCCTBU.InitialConcentration = 0;
        EC.Cyt.N.AJIYNVOQJR.InitialConcentration = 0;
        EC.Cyt.N.NRKRVULXIY.InitialConcentration = 0;
        EC.Cyt.N.DWPMBRWHEF.InitialConcentration = 0;
        EC.Cyt.N.VBOAMHRWEU.InitialConcentration = 0;
        EC.Cyt.N.XUKHUJBSKY.InitialConcentration = 1;
        EC.Cyt.N.LEADDBWRAG.InitialConcentration = 0;
        EC.Cyt.N.AGOBMLVSUW.InitialConcentration = 0;
        EC.Cyt.N.CVVQVNKOYS.InitialConcentration = 0;
        EC.Cyt.N.WXMNCUYFYD.InitialConcentration = 0;
        EC.Cyt.N.JKLDQAETKY.InitialConcentration = 1;
        EC.Cyt.N.QBUVTSDESA.InitialConcentration = 0;
        EC.Cyt.N.KYYFWUUFJR.InitialConcentration = 1.0E-4;
        EC.Cyt.N.LXLRJTCMCN.InitialConcentration = 0;
        EC.Cyt.N.LEPXNCOIXH.InitialConcentration = 0.1;
        EC.Cyt.N.LIKUEPJGFI.InitialConcentration = 0;
        EC.Cyt.N.VATMHXVNSV.InitialConcentration = 1;
        EC.Cyt.N.DWWTIFITMT.InitialConcentration = 0;
        EC.Cyt.N.TLGVYSXTLN.InitialConcentration = 1;
        real EC.Cyt.JNGRHJTOEM.Amount(t) umol;
        real EC.Cyt.JNGRHJTOEM.Concentration(t) uM;
        real EC.Cyt.JNGRHJTOEM.InitialConcentration uM;
        real EC.Cyt.NFKBIA_Txn.Flux(t) umol/sec;
        real EC.Cyt.NFKBIA_Txn.Vf umol/sec;
        real EC.Cyt.NFKBIA_Txn.Ki uM;
        real EC.Cyt.NFKBIA_Txn.Km_NFKBIAn uM;
        EC.Cyt.NFKBIA_Txn.Vf = 1.207/2.1;
        EC.Cyt.NFKBIA_Txn.Ki = 0.1;
        EC.Cyt.NFKBIA_Txn.Km_NFKBIAn = 1;
        EC.Cyt.NFKBIA_Txn.Flux = (EC.Cyt.NFKBIA_Txn.Vf*
(EC.Cyt.N.NFKB1n.Concentration^2))/(((EC.Cyt.NFKBIA_Txn.Km_NFKBIAn^2)*(1
+(EC.Cyt.N.XXMIPNQUOL.Concentration/EC.Cyt.NFKBIA_Txn.Ki)))
+(EC.Cyt.N.NFKB1n.Concentration^2));
        real EC.Cyt.PGRB_PG_Bd.Flux(t) umol/sec;
        real EC.Cyt.PGRB_PG_Bd.Kf 1/(uM*sec);
        real EC.Cyt.PGRB_PG_Bd.Kr 1/(uM*sec);
        EC.Cyt.PGRB_PG_Bd.Kf = 5;
        EC.Cyt.PGRB_PG_Bd.Flux =
(((EC.Cyt.PGRB_PG_Bd.Kf*EC.Cyt.IBUAUBLBAJ.Concentration)
*EC.Cyt.HXPQSSVMJO.Concentration)-
((EC.Cyt.PGRB_PG_Bd.Kr*EC.Cyt.GVEAOFSCUE.Concentration)
*EC.Cyt.FCMRIEXQWG.Concentration))*EC.Cyt.Vcyt;
        EC.Cyt.PGRB_PG_Bd.Kr = 0.0010;
        real EC.Cyt.mRNA_ESRn_Tr.Kf umol/(uM*sec);
        real EC.Cyt.mRNA_ESRn_Tr.Flux(t) umol/sec;
        real EC.Cyt.mRNA_ESRn_Tr.Kr umol/(uM*sec);
        EC.Cyt.mRNA_ESRn_Tr.Flux =
(EC.Cyt.mRNA_ESRn_Tr.Kf*EC.Cyt.N.MKNUYLQQGK.Concentration)-
(EC.Cyt.mRNA_ESRn_Tr.Kr*EC.Cyt.ESRc_mRNA.Concentration);
        EC.Cyt.mRNA_ESRn_Tr.Kf = 0.0010;
```

```
        EC.Cyt.mRNA_ESRn_Tr.Kr = 0;
        real EC.Cyt.PGRB_Frm.Flux(t) umol/sec;
        real EC.Cyt.PGRB_Frm.Kf 1/sec;
        EC.Cyt.PGRB_Frm.Kf = 0.1;
        EC.Cyt.PGRB_Frm.Flux = (EC.Cyt.PGRB_Frm.Kf*EC.Cyt.RFFXXGONQA.Concentration)
*EC.Cyt.Vcyt;
        real EC.Cyt.NFKBIAc_p_Dgr.Flux(t) umol/sec;
        real EC.Cyt.NFKBIAc_p_Dgr.Kf 1/sec;
        EC.Cyt.NFKBIAc_p_Dgr.Kf = 0.01;
        EC.Cyt.NFKBIAc_p_Dgr.Flux =
(EC.Cyt.NFKBIAc_p_Dgr.Kf*EC.Cyt.IHYPKQPWTX.Concentration)*EC.Cyt.Vcyt;
        real EC.Cyt.PGRA_Frm.Flux(t) umol/sec;
        real EC.Cyt.PGRA_Frm.Kf 1/sec;
        EC.Cyt.PGRA_Frm.Kf = 0.1;
        EC.Cyt.PGRA_Frm.Flux = (EC.Cyt.PGRA_Frm.Kf*EC.Cyt.XKVYWVGFII.Concentration)
*EC.Cyt.Vcyt;
        real EC.Cyt.PGRA_Trl.Flux(t) umol/sec;
        real EC.Cyt.PGRA_Trl.Kf 1/sec;
        EC.Cyt.PGRA_Trl.Kf = 0.01;
        EC.Cyt.PGRA_Trl.Flux = (EC.Cyt.PGRA_Trl.Kf*EC.Cyt.PGRAc_mRNA.Concentration)
*EC.Cyt.Vcyt;
        real EC.Cyt.CO_2_Rgn.Flux(t) umol/sec;
        real EC.Cyt.CO_2_Rgn.Kf 1/sec;
        EC.Cyt.CO_2_Rgn.Kf = 0.01;
        EC.Cyt.CO_2_Rgn.Flux = (EC.Cyt.CO_2_Rgn.Kf*EC.Cyt.LBXTYHSEFK.Concentration)
*EC.Cyt.Vcyt;
        real EC.Cyt.hsp90_1_Rgn.Flux(t) umol/sec;
        real EC.Cyt.hsp90_1_Rgn.Kf 1/sec;
        EC.Cyt.hsp90_1_Rgn.Kf = 0.1;
        EC.Cyt.hsp90_1_Rgn.Flux =
(EC.Cyt.hsp90_1_Rgn.Kf*EC.Cyt.VUVKKIUMSM.Concentration)*EC.Cyt.Vcyt;
        real EC.Cyt.PGRBmRNAc_Dgr.Flux(t) umol/sec;
        real EC.Cyt.PGRBmRNAc_Dgr.Kf 1/sec;
        EC.Cyt.PGRBmRNAc_Dgr.Kf = 1.0E-4;
        EC.Cyt.PGRBmRNAc_Dgr.Flux =
(EC.Cyt.PGRBmRNAc_Dgr.Kf*EC.Cyt.PGRBc_mRNA.Concentration)*EC.Cyt.Vcyt;
        real EC.Cyt.NFKB1c_Tr.Kf umol/(uM*sec);
        real EC.Cyt.NFKB1c_Tr.Flux(t) umol/sec;
        real EC.Cyt.NFKB1c_Tr.Kr umol/(uM*sec);
        EC.Cyt.NFKB1c_Tr.Flux = (EC.Cyt.NFKB1c_Tr.Kf*EC.Cyt.NFKB1c.Concentration)-
(EC.Cyt.NFKB1c_Tr.Kr*EC.Cyt.N.NFKB1n.Concentration);
        EC.Cyt.NFKB1c_Tr.Kf = 0.09;
        EC.Cyt.NFKB1c_Tr.Kr = 8.0E-5;
        real EC.Cyt.PG_Tr.Flux(t) umol/sec;
        real EC.Cyt.PG_Tr.Kf 1/sec;
        real EC.Cyt.PG_Tr.Kr 1/sec;
        EC.Cyt.PG_Tr.Kf = 20;
        EC.Cyt.PG_Tr.Flux = if (EC.Cyt.HXPQSSVMJO.Concentration<=0.5)
((((EC.Cyt.PG_Tr.Kf*EC.PG.Concentration)*0.2048)-
(EC.Cyt.PG_Tr.Kr*EC.Cyt.HXPQSSVMJO.Concentration))*EC.Cyt.Vcyt) else (0);
        EC.Cyt.PG_Tr.Kr = 0.0010;
        real EC.Cyt.CHUK_p_Dgr.Flux(t) umol/sec;
        real EC.Cyt.CHUK_p_Dgr.Kf 1/sec;
        EC.Cyt.CHUK_p_Dgr.Kf = 0.0072;
        EC.Cyt.CHUK_p_Dgr.Flux = (EC.Cyt.CHUK_p_Dgr.Kf*EC.Cyt.CHUK_p.Concentration)
*EC.Cyt.Vcyt;
        real EC.Cyt.hsp90_2_Rgn.Flux(t) umol/sec;
        real EC.Cyt.hsp90_2_Rgn.Kf 1/sec;
        EC.Cyt.hsp90_2_Rgn.Kf = 0.1;
        EC.Cyt.hsp90_2_Rgn.Flux =
(EC.Cyt.hsp90_2_Rgn.Kf*EC.Cyt.GVEAOFSCUE.Concentration)*EC.Cyt.Vcyt;
        real EC.Cyt.PGRB_hsp40_Bd.Flux(t) umol/sec;
        real EC.Cyt.PGRB_hsp40_Bd.Kf 1/(uM*sec);
        real EC.Cyt.PGRB_hsp40_Bd.Kr 1/sec;
```

```
        EC.Cyt.PGRB_hsp40_Bd.Kf = 0.01;
        EC.Cyt.PGRB_hsp40_Bd.Flux =
(((EC.Cyt.PGRB_hsp40_Bd.Kf*EC.Cyt.PGRB.Concentration)*EC.Cyt.BUKQBUVSPL.Concentration)-
(EC.Cyt.PGRB_hsp40_Bd.Kr*EC.Cyt.VESHECAPCO.Concentration))*EC.Cyt.Vcyt;
        EC.Cyt.PGRB_hsp40_Bd.Kr = 7.7E-4;
        real EC.Cyt.NFKBIAc_2_Dgr.Flux(t) umol/sec;
        real EC.Cyt.NFKBIAc_2_Dgr.Vf umol/sec;
        real EC.Cyt.NFKBIAc_2_Dgr.Km_CHUK_p_NFKBIAc uM;
        EC.Cyt.NFKBIAc_2_Dgr.Vf = 1.5;
        EC.Cyt.NFKBIAc_2_Dgr.Km_CHUK_p_NFKBIAc = 3.2;
        EC.Cyt.NFKBIAc_2_Dgr.Flux =
(EC.Cyt.NFKBIAc_2_Dgr.Vf*EC.Cyt.SFKBLJATXG.Concentration)/
(EC.Cyt.NFKBIAc_2_Dgr.Km_CHUK_p_NFKBIAc+EC.Cyt.SFKBLJATXG.Concentration);
        real EC.Cyt.ESR_Trl.Flux(t) umol/sec;
        real EC.Cyt.ESR_Trl.Kf 1/sec;
        EC.Cyt.ESR_Trl.Kf = 0.01;
        EC.Cyt.ESR_Trl.Flux = (EC.Cyt.ESR_Trl.Kf*EC.Cyt.ESRc_mRNA.Concentration)
*EC.Cyt.Vcyt;
        real EC.Cyt.PGRA_hsp40_Bd.Flux(t) umol/sec;
        real EC.Cyt.PGRA_hsp40_Bd.Kf 1/(uM*sec);
        real EC.Cyt.PGRA_hsp40_Bd.Kr 1/sec;
        EC.Cyt.PGRA_hsp40_Bd.Kf = 0.01;
        EC.Cyt.PGRA_hsp40_Bd.Flux =
(((EC.Cyt.PGRA_hsp40_Bd.Kf*EC.Cyt.BUKQBUVSPL.Concentration)*EC.Cyt.PGRA.Concentration)-
(EC.Cyt.PGRA_hsp40_Bd.Kr*EC.Cyt.IHRRYRSKWQ.Concentration))*EC.Cyt.Vcyt;
        EC.Cyt.PGRA_hsp40_Bd.Kr = 7.7E-4;
        real EC.Cyt.NFKBIAc_Tr.Kf umol/(uM*sec);
        real EC.Cyt.NFKBIAc_Tr.Flux(t) umol/sec;
        real EC.Cyt.NFKBIAc_Tr.Kr umol/(uM*sec);
        EC.Cyt.NFKBIAc_Tr.Flux = (EC.Cyt.NFKBIAc_Tr.Kf*EC.Cyt.NFKBIAc.Concentration)-
(EC.Cyt.NFKBIAc_Tr.Kr*EC.Cyt.N.VWLRCPRFDD.Concentration);
        EC.Cyt.NFKBIAc_Tr.Kf = 0.018;
        EC.Cyt.NFKBIAc_Tr.Kr = 0.012;
        real EC.Cyt.CO5_PGRA_Dis.Flux(t) umol/sec;
        real EC.Cyt.CO5_PGRA_Dis.Kf 1/sec;
        EC.Cyt.CO5_PGRA_Dis.Kf = 5;
        EC.Cyt.CO5_PGRA_Dis.Flux =
(EC.Cyt.CO5_PGRA_Dis.Kf*EC.Cyt.BDCIKSYKJY.Concentration)*EC.Cyt.Vcyt;
        real EC.Cyt.PGRB_Dgr.Flux(t) umol/sec;
        real EC.Cyt.PGRB_Dgr.Kf 1/sec;
        EC.Cyt.PGRB_Dgr.Kf = 1.0E-5;
        EC.Cyt.PGRB_Dgr.Flux = (EC.Cyt.PGRB_Dgr.Kf*EC.Cyt.PGRB.Concentration)
*EC.Cyt.Vcyt;
        real EC.Cyt.PGRA_Dgr.Flux(t) umol/sec;
        real EC.Cyt.PGRA_Dgr.Kf 1/sec;
        EC.Cyt.PGRA_Dgr.Kf = 1.0E-5;
        EC.Cyt.PGRA_Dgr.Flux = (EC.Cyt.PGRA_Dgr.Kf*EC.Cyt.PGRA.Concentration)
*EC.Cyt.Vcyt;
        real EC.Cyt.PGRB_Trl.Flux(t) umol/sec;
        real EC.Cyt.PGRB_Trl.Kf 1/sec;
        EC.Cyt.PGRB_Trl.Kf = 0.01;
        EC.Cyt.PGRB_Trl.Flux = (EC.Cyt.PGRB_Trl.Kf*EC.Cyt.PGRBc_mRNA.Concentration)
*EC.Cyt.Vcyt;
        real EC.Cyt.C02_ESR_Dis.Flux(t) umol/sec;
        real EC.Cyt.C02_ESR_Dis.Kf 1/sec;
        EC.Cyt.C02_ESR_Dis.Kf = 0.01;
        EC.Cyt.C02_ESR_Dis.Flux =
(EC.Cyt.C02_ESR_Dis.Kf*EC.Cyt.IIYBUWADRK.Concentration)*EC.Cyt.Vcyt;
        real EC.Cyt.mRNA_ESRc_Dgr.Flux(t) umol/sec;
        real EC.Cyt.mRNA_ESRc_Dgr.Kf 1/sec;
        EC.Cyt.mRNA_ESRc_Dgr.Kf = 1.0E-4;
        EC.Cyt.mRNA_ESRc_Dgr.Flux =
(EC.Cyt.mRNA_ESRc_Dgr.Kf*EC.Cyt.ESRc_mRNA.Concentration)*EC.Cyt.Vcyt;
        real EC.Cyt.CO_Hsp90_Bd.Flux(t) umol/sec;
```

```
        real EC.Cyt.CO_Hsp90_Bd.Kf 1/(uM*sec);
        real EC.Cyt.CO_Hsp90_Bd.Kr 1/sec;
        EC.Cyt.CO_Hsp90_Bd.Kf = 0.01;
        EC.Cyt.CO_Hsp90_Bd.Flux =
(((EC.Cyt.CO_Hsp90_Bd.Kf*EC.Cyt.SMMYREUOEE.Concentration)
*EC.Cyt.KMXEIYOWMQ.Concentration)-
(EC.Cyt.CO_Hsp90_Bd.Kr*EC.Cyt.JNGRHJTOEM.Concentration))*EC.Cyt.Vcyt;
        EC.Cyt.CO_Hsp90_Bd.Kr = 0.0010;
        real EC.Cyt.CHUK_p_NFKBIAc_NFKB1c_1_Bd.Flux(t) umol/sec;
        real EC.Cyt.CHUK_p_NFKBIAc_NFKB1c_1_Bd.Kf 1/(uM*sec);
        real EC.Cyt.CHUK_p_NFKBIAc_NFKB1c_1_Bd.Kr 1/sec;
        EC.Cyt.CHUK_p_NFKBIAc_NFKB1c_1_Bd.Kf = 0.5;
        EC.Cyt.CHUK_p_NFKBIAc_NFKB1c_1_Bd.Flux =
(((EC.Cyt.CHUK_p_NFKBIAc_NFKB1c_1_Bd.Kf*EC.Cyt.CHUK_p.Concentration)
*EC.Cyt.MEUHHCXDNY.Concentration)-
(EC.Cyt.CHUK_p_NFKBIAc_NFKB1c_1_Bd.Kr*EC.Cyt.QQDSAXKYSH.Concentration))*EC.Cyt.Vcyt;
        EC.Cyt.CHUK_p_NFKBIAc_NFKB1c_1_Bd.Kr = 5.0E-4;
        real EC.Cyt.NFKBIA_Trl.Flux(t) umol/sec;
        real EC.Cyt.NFKBIA_Trl.Vf umol/sec;
        real EC.Cyt.NFKBIA_Trl.Km_NFKBIAc uM;
        EC.Cyt.NFKBIA_Trl.Vf = 2.448;
        EC.Cyt.NFKBIA_Trl.Km_NFKBIAc = 9;
        EC.Cyt.NFKBIA_Trl.Flux =
(EC.Cyt.NFKBIA_Trl.Vf*EC.Cyt.N.PIBFOAUQHQ.Concentration)/(EC.Cyt.NFKBIA_Trl.Km_NFKBIAc
+EC.Cyt.N.PIBFOAUQHQ.Concentration);
        real EC.Cyt.C04_PGRB_Dis.Flux(t) umol/sec;
        real EC.Cyt.C04_PGRB_Dis.Kf 1/sec;
        EC.Cyt.C04_PGRB_Dis.Kf = 0.1;
        EC.Cyt.C04_PGRB_Dis.Flux =
(EC.Cyt.C04_PGRB_Dis.Kf*EC.Cyt.VXLSQVSTNA.Concentration)*EC.Cyt.Vcyt;
        real EC.Cyt.ESR_Frm.Flux(t) umol/sec;
        real EC.Cyt.ESR_Frm.Kf 1/sec;
        EC.Cyt.ESR_Frm.Kf = 0.1;
        EC.Cyt.ESR_Frm.Flux = (EC.Cyt.ESR_Frm.Kf*EC.Cyt.CUUPSCJFCK.Concentration)
*EC.Cyt.Vcyt;
        real EC.Cyt.PGRB_Tr.Kf umol/(uM*sec);
        real EC.Cyt.PGRB_Tr.Flux(t) umol/sec;
        real EC.Cyt.PGRB_Tr.Kr umol/(uM*sec);
        EC.Cyt.PGRB_Tr.Flux = (EC.Cyt.PGRB_Tr.Kf*EC.Cyt.FCMRIEXQWG.Concentration)-
(EC.Cyt.PGRB_Tr.Kr*EC.Cyt.N.XXMIPNQUOL.Concentration);
        EC.Cyt.PGRB_Tr.Kf = 0.33/60;
        EC.Cyt.PGRB_Tr.Kr = 0;
        real EC.Cyt.NFKBIAc_1_Dgr.Flux(t) umol/sec;
        real EC.Cyt.NFKBIAc_1_Dgr.Km_CHUK_p_NFKBIAc_NFkB1c uM;
        real EC.Cyt.NFKBIAc_1_Dgr.Vf umol/sec;
        EC.Cyt.NFKBIAc_1_Dgr.Km_CHUK_p_NFKBIAc_NFkB1c = 10;
        EC.Cyt.NFKBIAc_1_Dgr.Vf = 5;
        EC.Cyt.NFKBIAc_1_Dgr.Flux =
(EC.Cyt.NFKBIAc_1_Dgr.Vf*EC.Cyt.QQDSAXKYSH.Concentration)/
(EC.Cyt.NFKBIAc_1_Dgr.Km_CHUK_p_NFKBIAc_NFkB1c+EC.Cyt.QQDSAXKYSH.Concentration);
        real EC.Cyt.PGRA_CO1_Bd.Flux(t) umol/sec;
        real EC.Cyt.PGRA_CO1_Bd.Kf 1/(uM*sec);
        real EC.Cyt.PGRA_CO1_Bd.Kr 1/sec;
        EC.Cyt.PGRA_CO1_Bd.Kf = 0.14/60;
        EC.Cyt.PGRA_CO1_Bd.Flux =
(((EC.Cyt.PGRA_CO1_Bd.Kf*EC.Cyt.IHRRYRSKWQ.Concentration)
*EC.Cyt.JNGRHJTOEM.Concentration)-
(EC.Cyt.PGRA_CO1_Bd.Kr*EC.Cyt.BDCIKSYKJY.Concentration))*EC.Cyt.Vcyt;
        EC.Cyt.PGRA_CO1_Bd.Kr = 4.5E-5;
        real EC.Cyt.NFKBIAc_Dgr.Flux(t) umol/sec;
        real EC.Cyt.NFKBIAc_Dgr.Km_NFKBIAc_NFkB1c uM;
        real EC.Cyt.NFKBIAc_Dgr.Vf umol/sec;
        EC.Cyt.NFKBIAc_Dgr.Km_NFKBIAc_NFkB1c = 5;
        EC.Cyt.NFKBIAc_Dgr.Vf = 0.0080*10;
```

```
        EC.Cyt.NFKBIAc_Dgr.Flux =
(EC.Cyt.NFKBIAc_Dgr.Vf*EC.Cyt.MEUHHCXDNY.Concentration)/
(EC.Cyt.NFKBIAc_Dgr.Km_NFKBIAc_NFkB1c+EC.Cyt.MEUHHCXDNY.Concentration);
        real EC.Cyt.CO_3_Rgn.Flux(t) umol/sec;
        real EC.Cyt.CO_3_Rgn.Kf 1/sec;
        EC.Cyt.CO_3_Rgn.Kf = 0.01;
        EC.Cyt.CO_3_Rgn.Flux = (EC.Cyt.CO_3_Rgn.Kf*EC.Cyt.VCKNCEABFO.Concentration)
*EC.Cyt.Vcyt;
        real EC.Cyt.NFKBIAc_3_Dgr.Flux(t) umol/sec;
        real EC.Cyt.NFKBIAc_3_Dgr.Kf 1/sec;
        EC.Cyt.NFKBIAc_3_Dgr.Kf = 0.00675/10;
        EC.Cyt.NFKBIAc_3_Dgr.Flux =
(EC.Cyt.NFKBIAc_3_Dgr.Kf*EC.Cyt.NFKBIAc.Concentration)*EC.Cyt.Vcyt;
        real EC.Cyt.ESR1_ES_Bd.Flux(t) umol/sec;
        real EC.Cyt.ESR1_ES_Bd.Kf 1/(uM*sec);
        real EC.Cyt.ESR1_ES_Bd.Kr 1/(uM*sec);
        EC.Cyt.ESR1_ES_Bd.Kf = 5;
        EC.Cyt.ESR1_ES_Bd.Flux =
(((EC.Cyt.ESR1_ES_Bd.Kf*EC.Cyt.VQDPIGKQWC.Concentration)
*EC.Cyt.EFUYJJRUFC.Concentration)-
((EC.Cyt.ESR1_ES_Bd.Kr*EC.Cyt.VUVKKIUMSM.Concentration)
*EC.Cyt.YINGPPRWIO.Concentration))*EC.Cyt.Vcyt;
        EC.Cyt.ESR1_ES_Bd.Kr = 0.0010;
        real EC.Cyt.NFKBIAc_NFKB1c_Bd.Flux(t) umol/sec;
        real EC.Cyt.NFKBIAc_NFKB1c_Bd.Kf 1/(uM*sec);
        real EC.Cyt.NFKBIAc_NFKB1c_Bd.Kr 1/sec;
        EC.Cyt.NFKBIAc_NFKB1c_Bd.Kf = 5;
        EC.Cyt.NFKBIAc_NFKB1c_Bd.Flux =
(((EC.Cyt.NFKBIAc_NFKB1c_Bd.Kf*EC.Cyt.NFKBIAc.Concentration)
*EC.Cyt.NFKB1c.Concentration)-
(EC.Cyt.NFKBIAc_NFKB1c_Bd.Kr*EC.Cyt.MEUHHCXDNY.Concentration))*EC.Cyt.Vcyt;
        EC.Cyt.NFKBIAc_NFKB1c_Bd.Kr = 0.0050;
        real EC.Cyt.CO_1_Rgn.Flux(t) umol/sec;
        real EC.Cyt.CO_1_Rgn.Kf 1/sec;
        EC.Cyt.CO_1_Rgn.Kf = 0.01;
        EC.Cyt.CO_1_Rgn.Flux = (EC.Cyt.CO_1_Rgn.Kf*EC.Cyt.IVPLRKCCWI.Concentration)
*EC.Cyt.Vcyt;
        real EC.Cyt.PGRA_Tr.Kf umol/(uM*sec);
        real EC.Cyt.PGRA_Tr.Flux(t) umol/sec;
        real EC.Cyt.PGRA_Tr.Kr umol/(uM*sec);
        EC.Cyt.PGRA_Tr.Flux = (EC.Cyt.PGRA_Tr.Kf*EC.Cyt.TJMSEORQAV.Concentration)-
(EC.Cyt.PGRA_Tr.Kr*EC.Cyt.N.CAAYFXCTLG.Concentration);
        EC.Cyt.PGRA_Tr.Kf = 0.33/60;
        EC.Cyt.PGRA_Tr.Kr = 1.0E-4;
        real EC.Cyt.NFKBIA_mRNAn_Dgr.Flux(t) umol/sec;
        real EC.Cyt.NFKBIA_mRNAn_Dgr.Kf 1/sec;
        EC.Cyt.NFKBIA_mRNAn_Dgr.Kf = 0.0168;
        EC.Cyt.NFKBIA_mRNAn_Dgr.Flux =
(EC.Cyt.NFKBIA_mRNAn_Dgr.Kf*EC.Cyt.N.PIBFOAUQHQ.Concentration)*EC.Cyt.Vcyt;
        real EC.Cyt.ESR_Dgr.Flux(t) umol/sec;
        real EC.Cyt.ESR_Dgr.Kf 1/sec;
        EC.Cyt.ESR_Dgr.Kf = 1.0E-5;
        EC.Cyt.ESR_Dgr.Flux = (EC.Cyt.ESR_Dgr.Kf*EC.Cyt.ESR1.Concentration)*EC.Cyt.Vcyt;
        real EC.Cyt.PGRA_PG_Bd.Flux(t) umol/sec;
        real EC.Cyt.PGRA_PG_Bd.Kf 1/(uM*sec);
        real EC.Cyt.PGRA_PG_Bd.Kr 1/(uM*sec);
        EC.Cyt.PGRA_PG_Bd.Kf = 5;
        EC.Cyt.PGRA_PG_Bd.Flux =
(((EC.Cyt.PGRA_PG_Bd.Kf*EC.Cyt.HXPQSSVMJO.Concentration)
*EC.Cyt.JWYDCVVNFY.Concentration)-
((EC.Cyt.PGRA_PG_Bd.Kr*EC.Cyt.TJMSEORQAV.Concentration)
*EC.Cyt.TBJMXSSCJU.Concentration))*EC.Cyt.Vcyt;
        EC.Cyt.PGRA_PG_Bd.Kr = 0.0010;
        real EC.Cyt.ESR1_CO1_Bd.Flux(t) umol/sec;
```

```
        real EC.Cyt.ESR1_CO1_Bd.Kf 1/(uM*sec);
        real EC.Cyt.ESR1_CO1_Bd.Kr 1/sec;
        EC.Cyt.ESR1_CO1_Bd.Kf = 0.14/60;
        EC.Cyt.ESR1_CO1_Bd.Flux =
(((EC.Cyt.ESR1_CO1_Bd.Kf*EC.Cyt.JNGRHJTOEM.Concentration)*EC.Cyt.ESR1.Concentration)-
(EC.Cyt.ESR1_CO1_Bd.Kr*EC.Cyt.IIYBUWADRK.Concentration))*EC.Cyt.Vcyt;
        EC.Cyt.ESR1_CO1_Bd.Kr = 4.5E-5;
        real EC.Cyt.Hsp90_Dis.Flux(t) umol/sec;
        real EC.Cyt.Hsp90_Dis.Kf 1/sec;
        real EC.Cyt.Hsp90_Dis.Kr 1/sec;
        EC.Cyt.Hsp90_Dis.Kf = 0.05;
        EC.Cyt.Hsp90_Dis.Flux =
((EC.Cyt.Hsp90_Dis.Kf*EC.Cyt.HSP90AA1_pool.Concentration)-
(EC.Cyt.Hsp90_Dis.Kr*EC.Cyt.SMMYREUOEE.Concentration))*EC.Cyt.Vcyt;
        EC.Cyt.Hsp90_Dis.Kr = 0.01;
        real EC.Cyt.PGRB_CO1_Bd.Flux(t) umol/sec;
        real EC.Cyt.PGRB_CO1_Bd.Kf 1/(uM*sec);
        real EC.Cyt.PGRB_CO1_Bd.Kr 1/sec;
        EC.Cyt.PGRB_CO1_Bd.Kf = 0.14/60;
        EC.Cyt.PGRB_CO1_Bd.Flux =
(((EC.Cyt.PGRB_CO1_Bd.Kf*EC.Cyt.VESHECAPCO.Concentration)
*EC.Cyt.JNGRHJTOEM.Concentration)-
(EC.Cyt.PGRB_CO1_Bd.Kr*EC.Cyt.VXLSQVSTNA.Concentration))*EC.Cyt.Vcyt;
        EC.Cyt.PGRB_CO1_Bd.Kr = 4.5E-5;
        real EC.Cyt.CHUK_p_NFKBIAc_NFKB1c_2_Bd.Flux(t) umol/sec;
        real EC.Cyt.CHUK_p_NFKBIAc_NFKB1c_2_Bd.Kf 1/(uM*sec);
        real EC.Cyt.CHUK_p_NFKBIAc_NFKB1c_2_Bd.Kr 1/sec;
        EC.Cyt.CHUK_p_NFKBIAc_NFKB1c_2_Bd.Kf = 0.185;
        EC.Cyt.CHUK_p_NFKBIAc_NFKB1c_2_Bd.Flux =
(((EC.Cyt.CHUK_p_NFKBIAc_NFKB1c_2_Bd.Kf*EC.Cyt.NFKB1c.Concentration)
*EC.Cyt.SFKBLJATXG.Concentration)-
(EC.Cyt.CHUK_p_NFKBIAc_NFKB1c_2_Bd.Kr*EC.Cyt.QQDSAXKYSH.Concentration))*EC.Cyt.Vcyt;
        EC.Cyt.CHUK_p_NFKBIAc_NFKB1c_2_Bd.Kr = 0.00125;
        real EC.Cyt.CO_ATP_Bd.Flux(t) umol/sec;
        real EC.Cyt.CO_ATP_Bd.Kf 1/(uM*sec);
        real EC.Cyt.CO_ATP_Bd.Kr 1/sec;
        EC.Cyt.CO_ATP_Bd.Kf = 1.0E-4;
        EC.Cyt.CO_ATP_Bd.Flux = (((EC.Cyt.CO_ATP_Bd.Kf*EC.Cyt.ATP.Concentration)
*EC.Cyt.CO.Concentration)-(EC.Cyt.CO_ATP_Bd.Kr*EC.Cyt.KMXEIYOWMQ.Concentration))
*EC.Cyt.Vcyt;
        EC.Cyt.CO_ATP_Bd.Kr = 0.01;
        real EC.Cyt.PGRBmRNA_Tr.Kf umol/(uM*sec);
        real EC.Cyt.PGRBmRNA_Tr.Flux(t) umol/sec;
        real EC.Cyt.PGRBmRNA_Tr.Kr umol/(uM*sec);
        EC.Cyt.PGRBmRNA_Tr.Flux =
(EC.Cyt.PGRBmRNA_Tr.Kf*EC.Cyt.N.LSPVTOVYYU.Concentration)-
(EC.Cyt.PGRBmRNA_Tr.Kr*EC.Cyt.PGRBc_mRNA.Concentration);
        EC.Cyt.PGRBmRNA_Tr.Kf = 0.0010;
        EC.Cyt.PGRBmRNA_Tr.Kr = 0;
        real EC.Cyt.hsp90_3_Rgn.Flux(t) umol/sec;
        real EC.Cyt.hsp90_3_Rgn.Kf 1/sec;
        EC.Cyt.hsp90_3_Rgn.Kf = 0.1;
        EC.Cyt.hsp90_3_Rgn.Flux =
(EC.Cyt.hsp90_3_Rgn.Kf*EC.Cyt.TBJMXSSCJU.Concentration)*EC.Cyt.Vcyt;
        real EC.Cyt.ES_Tr.Flux(t) umol/sec;
        real EC.Cyt.ES_Tr.Kf 1/sec;
        real EC.Cyt.ES_Tr.Kr 1/sec;
        EC.Cyt.ES_Tr.Kf = 10;
        EC.Cyt.ES_Tr.Flux = if (EC.Cyt.EFUYJJRUFC.Concentration<=0.5)
((((EC.Cyt.ES_Tr.Kf*EC.ES.Concentration)*0.2048)-
(EC.Cyt.ES_Tr.Kr*EC.Cyt.EFUYJJRUFC.Concentration))*EC.Cyt.Vcyt) else (0);
        EC.Cyt.ES_Tr.Kr = 0.0010;
        real EC.Cyt.PGRAmRNA_Tr.Kf umol/(uM*sec);
        real EC.Cyt.PGRAmRNA_Tr.Flux(t) umol/sec;
```

```
        real EC.Cyt.PGRAmRNA_Tr.Kr umol/(uM*sec);
        EC.Cyt.PGRAmRNA_Tr.Flux =
(EC.Cyt.PGRAmRNA_Tr.Kf*EC.Cyt.N.KYYFWUUFJR.Concentration)-
(EC.Cyt.PGRAmRNA_Tr.Kr*EC.Cyt.PGRAc_mRNA.Concentration);
        EC.Cyt.PGRAmRNA_Tr.Kf = 0.0010;
        EC.Cyt.PGRAmRNA_Tr.Kr = 0;
        real EC.Cyt.NFKBIA_1_Txn.Flux(t) umol/sec;
        real EC.Cyt.NFKBIA_1_Txn.C;
        EC.Cyt.NFKBIA_1_Txn.C = 9.214E-5;
        EC.Cyt.NFKBIA_1_Txn.Flux = EC.Cyt.NFKBIA_1_Txn.C;
        real EC.Cyt.ESR1_Tr.Kf umol/(uM*sec);
        real EC.Cyt.ESR1_Tr.Flux(t) umol/sec;
        real EC.Cyt.ESR1_Tr.Kr umol/(uM*sec);
        EC.Cyt.ESR1_Tr.Flux = (EC.Cyt.ESR1_Tr.Kf*EC.Cyt.YINGPPRWIO.Concentration)-
(EC.Cyt.ESR1_Tr.Kr*EC.Cyt.N.LXLRJTCMCN.Concentration);
        EC.Cyt.ESR1_Tr.Kf = 0.33/60;
        EC.Cyt.ESR1_Tr.Kr = 0;
        real EC.Cyt.PGRmRNAc_Dgr.Flux(t) umol/sec;
        real EC.Cyt.PGRmRNAc_Dgr.Kf 1/sec;
        EC.Cyt.PGRmRNAc_Dgr.Kf = 1.0E-4;
        EC.Cyt.PGRmRNAc_Dgr.Flux =
(EC.Cyt.PGRmRNAc_Dgr.Kf*EC.Cyt.PGRAc_mRNA.Concentration)*EC.Cyt.Vcyt;
        real EC.Cyt.CHUK_p_NFKBIAc_Bd.Flux(t) umol/sec;
        real EC.Cyt.CHUK_p_NFKBIAc_Bd.Kf 1/(uM*sec);
        real EC.Cyt.CHUK_p_NFKBIAc_Bd.Kr 1/sec;
        EC.Cyt.CHUK_p_NFKBIAc_Bd.Kf = 2.25;
        EC.Cyt.CHUK_p_NFKBIAc_Bd.Flux =
(((EC.Cyt.CHUK_p_NFKBIAc_Bd.Kf*EC.Cyt.CHUK_p.Concentration)
*EC.Cyt.NFKBIAc.Concentration)-
(EC.Cyt.CHUK_p_NFKBIAc_Bd.Kr*EC.Cyt.SFKBLJATXG.Concentration))*EC.Cyt.Vcyt;
        EC.Cyt.CHUK_p_NFKBIAc_Bd.Kr = 0.00125;
        EC.Cyt.Vcyt = 1.66;
        EC.Cyt.Volume = 1.66;
        EC.Cyt.ESRc_mRNA.InitialConcentration = 0;
        EC.Cyt.VQDPIGKQWC.InitialConcentration = 0;
        EC.Cyt.BDCIKSYKJY.InitialConcentration = 0;
        EC.Cyt.NFKBIAc.InitialConcentration = 0.372;
        EC.Cyt.TJMSEORQAV.InitialConcentration = 0;
        EC.Cyt.WSQTUBSXTX.Concentration = 0;
        EC.Cyt.TBJMXSSCJU.InitialConcentration = 0;
        EC.Cyt.YINGPPRWIO.InitialConcentration = 0;
        EC.Cyt.VUVKKIUMSM.InitialConcentration = 0;
        EC.Cyt.RFFXXGONQA.InitialConcentration = 0;
        EC.Cyt.FCMRIEXQWG.InitialConcentration = 0;
        EC.Cyt.CHUK_p.InitialConcentration = 0.0010;
        EC.Cyt.ADP.InitialConcentration = 0;
        EC.Cyt.SMMYREUOEE.InitialConcentration = 0;
        EC.Cyt.NFKB1c.InitialConcentration = 0.003053;
        EC.Cyt.MEUHHCXDNY.InitialConcentration = 0.09826;
        EC.Cyt.PGRB.InitialConcentration = 0.04;
        EC.Cyt.XKVYWVGFII.InitialConcentration = 0;
        EC.Cyt.KMXEIYOWMQ.InitialConcentration = 0;
        EC.Cyt.IVPLRKCCWI.InitialConcentration = 0;
        EC.Cyt.LBXTYHSEFK.InitialConcentration = 0;
        EC.Cyt.SFKBLJATXG.InitialConcentration = 0;
        EC.Cyt.PGRBc_mRNA.InitialConcentration = 0;
        EC.Cyt.PGRA.InitialConcentration = 0.04;
        EC.Cyt.VXLSQVSTNA.InitialConcentration = 0;
        EC.Cyt.HSP90AA1_pool.InitialConcentration = 20;
        EC.Cyt.EFUYJJRUFC.InitialConcentration = 0;
        EC.Cyt.BUKQBUVSPL.InitialConcentration = 10;
        EC.Cyt.IBUAUBLBAJ.InitialConcentration = 0;
        EC.Cyt.QQDSAXKYSH.InitialConcentration = 0;
        EC.Cyt.ATP.InitialConcentration = 5000;
```

```
        EC.Cyt.JWYDCVVNFY.InitialConcentration = 0;
        EC.Cyt.ESR1.InitialConcentration = 0.01;
        EC.Cyt.IHYPKQPWTX.InitialConcentration = 0;
        EC.Cyt.GVEAOFSCUE.InitialConcentration = 0;
        EC.Cyt.PGRAc_mRNA.InitialConcentration = 0;
        EC.Cyt.VESHECAPCO.InitialConcentration = 0;
        EC.Cyt.CUUPSCJFCK.InitialConcentration = 0;
        EC.Cyt.IHRRYRSKWQ.InitialConcentration = 0;
        EC.Cyt.CO.InitialConcentration = 10;
        EC.Cyt.VCKNCEABFO.InitialConcentration = 0;
        EC.Cyt.HXPQSSVMJO.InitialConcentration = 0;
        EC.Cyt.IIYBUWADRK.InitialConcentration = 0;
        EC.Cyt.JNGRHJTOEM.InitialConcentration = 0;
        EC.Cyt.N.FAAYBECMNJ.Concentration = EC.Cyt.N.FAAYBECMNJ.Amount/EC.Cyt.N.Volume;
        when (t=t.min) EC.Cyt.N.FAAYBECMNJ.Amount =
EC.Cyt.N.FAAYBECMNJ.InitialConcentration*EC.Cyt.N.Volume;
        EC.Cyt.N.FAAYBECMNJ.Amount:t = EC.Cyt.N.TP3_PCAF_Bd.Flux-
EC.Cyt.N.TP4_TRAP_Bd.Flux;
        EC.Cyt.N.PIBFOAUQHQ.Concentration = EC.Cyt.N.PIBFOAUQHQ.Amount/EC.Cyt.N.Volume;
        when (t=t.min) EC.Cyt.N.PIBFOAUQHQ.Amount =
EC.Cyt.N.PIBFOAUQHQ.InitialConcentration*EC.Cyt.N.Volume;
        EC.Cyt.N.PIBFOAUQHQ.Amount:t = (EC.Cyt.NFKBIA_Txn.Flux-
EC.Cyt.NFKBIA_mRNAn_Dgr.Flux)+EC.Cyt.NFKBIA_1_Txn.Flux;
        EC.Cyt.N.MTACYBTIEX.Concentration = EC.Cyt.N.MTACYBTIEX.Amount/EC.Cyt.N.Volume;
        when (t=t.min) EC.Cyt.N.MTACYBTIEX.Amount =
EC.Cyt.N.MTACYBTIEX.InitialConcentration*EC.Cyt.N.Volume;
        EC.Cyt.N.MTACYBTIEX.Amount:t = EC.Cyt.N.TC4_TRAP_Bd.Flux;
        EC.Cyt.N.PWJCQEHQNG.Concentration = EC.Cyt.N.PWJCQEHQNG.Amount/EC.Cyt.N.Volume;
        when (t=t.min) EC.Cyt.N.PWJCQEHQNG.Amount =
EC.Cyt.N.PWJCQEHQNG.InitialConcentration*EC.Cyt.N.Volume;
        EC.Cyt.N.PWJCQEHQNG.Amount:t = (-EC.Cyt.N.TC1_NCOA_AC.Flux)
+EC.Cyt.N.ESR_ERE_Bd.Flux;
        EC.Cyt.N.DONQANHYGT.Concentration = EC.Cyt.N.DONQANHYGT.Amount/EC.Cyt.N.Volume;
        when (t=t.min) EC.Cyt.N.DONQANHYGT.Amount =
EC.Cyt.N.DONQANHYGT.InitialConcentration*EC.Cyt.N.Volume;
        EC.Cyt.N.DONQANHYGT.Amount:t = (-EC.Cyt.N.TC2_CBP_Bd.Flux)
+EC.Cyt.N.TC1_NCOA_AC.Flux;
        EC.Cyt.N.LSPVTOVYYU.Concentration = EC.Cyt.N.LSPVTOVYYU.Amount/EC.Cyt.N.Volume;
        when (t=t.min) EC.Cyt.N.LSPVTOVYYU.Amount =
EC.Cyt.N.LSPVTOVYYU.InitialConcentration*EC.Cyt.N.Volume;
        EC.Cyt.N.LSPVTOVYYU.Amount:t = ((-EC.Cyt.PGRBmRNA_Tr.Flux)-
EC.Cyt.N.PGRmRNAn_Dgr.Flux)+EC.Cyt.N.PGRBmRNA_Txn.Flux;
        EC.Cyt.N.MDTAOIEDKY.Concentration = EC.Cyt.N.MDTAOIEDKY.Amount/EC.Cyt.N.Volume;
        when (t=t.min) EC.Cyt.N.MDTAOIEDKY.Amount =
EC.Cyt.N.MDTAOIEDKY.InitialConcentration*EC.Cyt.N.Volume;
        EC.Cyt.N.MDTAOIEDKY.Amount:t = EC.Cyt.N.TPA3_PCAF_Bd.Flux-
EC.Cyt.N.TPA4_TRAP_Bd.Flux;
        EC.Cyt.N.NOCWORPSMX.Concentration = EC.Cyt.N.NOCWORPSMX.Amount/EC.Cyt.N.Volume;
        when (t=t.min) EC.Cyt.N.NOCWORPSMX.Amount =
EC.Cyt.N.NOCWORPSMX.InitialConcentration*EC.Cyt.N.Volume;
        EC.Cyt.N.NOCWORPSMX.Amount:t = EC.Cyt.N.ES_ESR1_Di.Flux-
EC.Cyt.N.ESR_ERE_Bd.Flux;
        EC.Cyt.N.QKSUBHODFF.Concentration = EC.Cyt.N.QKSUBHODFF.Amount/EC.Cyt.N.Volume;
        when (t=t.min) EC.Cyt.N.QKSUBHODFF.Amount =
EC.Cyt.N.QKSUBHODFF.InitialConcentration*EC.Cyt.N.Volume;
        EC.Cyt.N.QKSUBHODFF.Amount:t = ((-EC.Cyt.N.PGRA_NCOA_AC.Flux)-
EC.Cyt.N.TC1_NCOA_AC.Flux)-EC.Cyt.N.PGRB_NCOA_AC.Flux;
        EC.Cyt.N.HIIDJNHNHE.Concentration = EC.Cyt.N.HIIDJNHNHE.Amount/EC.Cyt.N.Volume;
        when (t=t.min) EC.Cyt.N.HIIDJNHNHE.Amount =
EC.Cyt.N.HIIDJNHNHE.InitialConcentration*EC.Cyt.N.Volume;
        EC.Cyt.N.HIIDJNHNHE.Amount:t = -EC.Cyt.N.ESR_ERE_Bd.Flux;
        EC.Cyt.N.CAAYFXCTLG.Concentration = EC.Cyt.N.CAAYFXCTLG.Amount/EC.Cyt.N.Volume;
        when (t=t.min) EC.Cyt.N.CAAYFXCTLG.Amount =
EC.Cyt.N.CAAYFXCTLG.InitialConcentration*EC.Cyt.N.Volume;
```

```
        EC.Cyt.N.CAAYFXCTLG.Amount:t = EC.Cyt.PGRA_Tr.Flux+(EC.Cyt.N.PG_PGRA_Di.Flux*
(-2));
        EC.Cyt.N.VWLRCPRFDD.Concentration = EC.Cyt.N.VWLRCPRFDD.Amount/EC.Cyt.N.Volume;
        when (t=t.min) EC.Cyt.N.VWLRCPRFDD.Amount =
EC.Cyt.N.VWLRCPRFDD.InitialConcentration*EC.Cyt.N.Volume;
        EC.Cyt.N.VWLRCPRFDD.Amount:t = EC.Cyt.NFKBIAc_Tr.Flux-
EC.Cyt.N.NFKBIAn_NFKB1n_Bd.Flux;
        EC.Cyt.N.NFKB1n.Concentration = EC.Cyt.N.NFKB1n.Amount/EC.Cyt.N.Volume;
        when (t=t.min) EC.Cyt.N.NFKB1n.Amount =
EC.Cyt.N.NFKB1n.InitialConcentration*EC.Cyt.N.Volume;
        EC.Cyt.N.NFKB1n.Amount:t = EC.Cyt.NFKB1c_Tr.Flux-
EC.Cyt.N.NFKBIAn_NFKB1n_Bd.Flux;
        EC.Cyt.N.XXMIPNQUOL.Concentration = EC.Cyt.N.XXMIPNQUOL.Amount/EC.Cyt.N.Volume;
        when (t=t.min) EC.Cyt.N.XXMIPNQUOL.Amount =
EC.Cyt.N.XXMIPNQUOL.InitialConcentration*EC.Cyt.N.Volume;
        EC.Cyt.N.XXMIPNQUOL.Amount:t = EC.Cyt.PGRB_Tr.Flux+(EC.Cyt.N.PG_PGRB_Di.Flux*
(-2));
        EC.Cyt.N.MKNUYLQQGK.Concentration = EC.Cyt.N.MKNUYLQQGK.Amount/EC.Cyt.N.Volume;
        when (t=t.min) EC.Cyt.N.MKNUYLQQGK.Amount =
EC.Cyt.N.MKNUYLQQGK.InitialConcentration*EC.Cyt.N.Volume;
        EC.Cyt.N.MKNUYLQQGK.Amount:t = ((-EC.Cyt.mRNA_ESRn_Tr.Flux)
+EC.Cyt.N.ESRmRNA_Txn.Flux)-EC.Cyt.N.ESRmRNAn_Dgr.Flux;
        EC.Cyt.N.NKGFVKNAGT.Concentration = EC.Cyt.N.NKGFVKNAGT.Amount/EC.Cyt.N.Volume;
        when (t=t.min) EC.Cyt.N.NKGFVKNAGT.Amount =
EC.Cyt.N.NKGFVKNAGT.InitialConcentration*EC.Cyt.N.Volume;
        EC.Cyt.N.NKGFVKNAGT.Amount:t = EC.Cyt.N.PGRA_NCOA_AC.Flux-
EC.Cyt.N.TPA2_CBP_Bd.Flux;
        EC.Cyt.N.IFRVPCCTBU.Concentration = EC.Cyt.N.IFRVPCCTBU.Amount/EC.Cyt.N.Volume;
        when (t=t.min) EC.Cyt.N.IFRVPCCTBU.Amount =
EC.Cyt.N.IFRVPCCTBU.InitialConcentration*EC.Cyt.N.Volume;
        EC.Cyt.N.IFRVPCCTBU.Amount:t = (-EC.Cyt.N.TP3_PCAF_Bd.Flux)
+EC.Cyt.N.TP2_CBP_Bd.Flux;
        EC.Cyt.N.AJIYNVOQJR.Concentration = EC.Cyt.N.AJIYNVOQJR.Amount/EC.Cyt.N.Volume;
        when (t=t.min) EC.Cyt.N.AJIYNVOQJR.Amount =
EC.Cyt.N.AJIYNVOQJR.InitialConcentration*EC.Cyt.N.Volume;
        EC.Cyt.N.AJIYNVOQJR.Amount:t = EC.Cyt.N.TP4_TRAP_Bd.Flux;
        EC.Cyt.N.NRKRVULXIY.Concentration = EC.Cyt.N.NRKRVULXIY.Amount/EC.Cyt.N.Volume;
        when (t=t.min) EC.Cyt.N.NRKRVULXIY.Amount =
EC.Cyt.N.NRKRVULXIY.InitialConcentration*EC.Cyt.N.Volume;
        EC.Cyt.N.NRKRVULXIY.Amount:t = EC.Cyt.N.TPA2_CBP_Bd.Flux-
EC.Cyt.N.TPA3_PCAF_Bd.Flux;
        EC.Cyt.N.DWPMBRWHEF.Concentration = EC.Cyt.N.DWPMBRWHEF.Amount/EC.Cyt.N.Volume;
        when (t=t.min) EC.Cyt.N.DWPMBRWHEF.Amount =
EC.Cyt.N.DWPMBRWHEF.InitialConcentration*EC.Cyt.N.Volume;
        EC.Cyt.N.DWPMBRWHEF.Amount:t = (-EC.Cyt.N.TC4_TRAP_Bd.Flux)
+EC.Cyt.N.TC3_BRG_Bd.Flux;
        EC.Cyt.N.VBOAMHRWEU.Concentration = EC.Cyt.N.VBOAMHRWEU.Amount/EC.Cyt.N.Volume;
        when (t=t.min) EC.Cyt.N.VBOAMHRWEU.Amount =
EC.Cyt.N.VBOAMHRWEU.InitialConcentration*EC.Cyt.N.Volume;
        EC.Cyt.N.VBOAMHRWEU.Amount:t = EC.Cyt.N.PGRB_PRE_Bd.Flux-
EC.Cyt.N.PGRB_NCOA_AC.Flux;
        EC.Cyt.N.XUKHUJBSKY.Concentration = EC.Cyt.N.XUKHUJBSKY.Amount/EC.Cyt.N.Volume;
        when (t=t.min) EC.Cyt.N.XUKHUJBSKY.Amount =
EC.Cyt.N.XUKHUJBSKY.InitialConcentration*EC.Cyt.N.Volume;
        EC.Cyt.N.XUKHUJBSKY.Amount:t = ((-EC.Cyt.N.TC2_CBP_Bd.Flux)-
EC.Cyt.N.TPA2_CBP_Bd.Flux)-EC.Cyt.N.TP2_CBP_Bd.Flux;
        EC.Cyt.N.LEADDBWRAG.Concentration = EC.Cyt.N.LEADDBWRAG.Amount/EC.Cyt.N.Volume;
        when (t=t.min) EC.Cyt.N.LEADDBWRAG.Amount =
EC.Cyt.N.LEADDBWRAG.InitialConcentration*EC.Cyt.N.Volume;
        EC.Cyt.N.LEADDBWRAG.Amount:t = (-EC.Cyt.N.NFKBIAn_NFKB1n_Tr.Flux)
+EC.Cyt.N.NFKBIAn_NFKB1n_Bd.Flux;
        EC.Cyt.N.AGOBMLVSUW.Concentration = EC.Cyt.N.AGOBMLVSUW.Amount/EC.Cyt.N.Volume;
        when (t=t.min) EC.Cyt.N.AGOBMLVSUW.Amount =
EC.Cyt.N.AGOBMLVSUW.InitialConcentration*EC.Cyt.N.Volume;
```

```
        EC.Cyt.N.AGOBMLVSUW.Amount:t = (-EC.Cyt.N.PGRA_NCOA_AC.Flux)
+EC.Cyt.N.PGRA_PRE_Bd.Flux;
        EC.Cyt.N.CVVQVNKOYS.Concentration = EC.Cyt.N.CVVQVNKOYS.Amount/EC.Cyt.N.Volume;
        when (t=t.min) EC.Cyt.N.CVVQVNKOYS.Amount =
EC.Cyt.N.CVVQVNKOYS.InitialConcentration*EC.Cyt.N.Volume;
        EC.Cyt.N.CVVQVNKOYS.Amount:t = EC.Cyt.N.PG_PGRA_Di.Flux-
EC.Cyt.N.PGRA_PRE_Bd.Flux;
        EC.Cyt.N.WXMNCUYFYD.Concentration = EC.Cyt.N.WXMNCUYFYD.Amount/EC.Cyt.N.Volume;
        when (t=t.min) EC.Cyt.N.WXMNCUYFYD.Amount =
EC.Cyt.N.WXMNCUYFYD.InitialConcentration*EC.Cyt.N.Volume;
        EC.Cyt.N.WXMNCUYFYD.Amount:t = (-EC.Cyt.N.PGRB_PRE_Bd.Flux)
+EC.Cyt.N.PG_PGRB_Di.Flux;
        EC.Cyt.N.JKLDQAETKY.Concentration = EC.Cyt.N.JKLDQAETKY.Amount/EC.Cyt.N.Volume;
        when (t=t.min) EC.Cyt.N.JKLDQAETKY.Amount =
EC.Cyt.N.JKLDQAETKY.InitialConcentration*EC.Cyt.N.Volume;
        EC.Cyt.N.JKLDQAETKY.Amount:t = ((-EC.Cyt.N.TC4_TRAP_Bd.Flux)-
EC.Cyt.N.TP4_TRAP_Bd.Flux)-EC.Cyt.N.TPA4_TRAP_Bd.Flux;
        EC.Cyt.N.QBUVTSDESA.Concentration = EC.Cyt.N.QBUVTSDESA.Amount/EC.Cyt.N.Volume;
        when (t=t.min) EC.Cyt.N.QBUVTSDESA.Amount =
EC.Cyt.N.QBUVTSDESA.InitialConcentration*EC.Cyt.N.Volume;
        EC.Cyt.N.QBUVTSDESA.Amount:t = EC.Cyt.N.TPA4_TRAP_Bd.Flux;
        EC.Cyt.N.KYYFWUUFJR.Concentration = EC.Cyt.N.KYYFWUUFJR.Amount/EC.Cyt.N.Volume;
        when (t=t.min) EC.Cyt.N.KYYFWUUFJR.Amount =
EC.Cyt.N.KYYFWUUFJR.InitialConcentration*EC.Cyt.N.Volume;
        EC.Cyt.N.KYYFWUUFJR.Amount:t = ((-EC.Cyt.PGRAmRNA_Tr.Flux)-
EC.Cyt.N.PGRAmRNAn_Dgr.Flux)+EC.Cyt.N.PGRAmRNA_Txn.Flux;
        EC.Cyt.N.LXLRJTCMCN.Concentration = EC.Cyt.N.LXLRJTCMCN.Amount/EC.Cyt.N.Volume;
        when (t=t.min) EC.Cyt.N.LXLRJTCMCN.Amount =
EC.Cyt.N.LXLRJTCMCN.InitialConcentration*EC.Cyt.N.Volume;
        EC.Cyt.N.LXLRJTCMCN.Amount:t = EC.Cyt.ESR1_Tr.Flux+(EC.Cyt.N.ES_ESR1_Di.Flux*
(-2));
        EC.Cyt.N.LEPXNCOIXH.Concentration = EC.Cyt.N.LEPXNCOIXH.Amount/EC.Cyt.N.Volume;
        when (t=t.min) EC.Cyt.N.LEPXNCOIXH.Amount =
EC.Cyt.N.LEPXNCOIXH.InitialConcentration*EC.Cyt.N.Volume;
        EC.Cyt.N.LEPXNCOIXH.Amount:t = (-EC.Cyt.N.PGRB_PRE_Bd.Flux)-
EC.Cyt.N.PGRA_PRE_Bd.Flux;
        EC.Cyt.N.LIKUEPJGFI.Concentration = EC.Cyt.N.LIKUEPJGFI.Amount/EC.Cyt.N.Volume;
        when (t=t.min) EC.Cyt.N.LIKUEPJGFI.Amount =
EC.Cyt.N.LIKUEPJGFI.InitialConcentration*EC.Cyt.N.Volume;
        EC.Cyt.N.LIKUEPJGFI.Amount:t = EC.Cyt.N.TC2_CBP_Bd.Flux-
EC.Cyt.N.TC3_BRG_Bd.Flux;
        EC.Cyt.N.VATMHXVNSV.Concentration = EC.Cyt.N.VATMHXVNSV.Amount/EC.Cyt.N.Volume;
        when (t=t.min) EC.Cyt.N.VATMHXVNSV.Amount =
EC.Cyt.N.VATMHXVNSV.InitialConcentration*EC.Cyt.N.Volume;
        EC.Cyt.N.VATMHXVNSV.Amount:t = (-EC.Cyt.N.TP3_PCAF_Bd.Flux)-
EC.Cyt.N.TPA3_PCAF_Bd.Flux;
        EC.Cyt.N.DWWTIFITMT.Concentration = EC.Cyt.N.DWWTIFITMT.Amount/EC.Cyt.N.Volume;
        when (t=t.min) EC.Cyt.N.DWWTIFITMT.Amount =
EC.Cyt.N.DWWTIFITMT.InitialConcentration*EC.Cyt.N.Volume;
        EC.Cyt.N.DWWTIFITMT.Amount:t = (-EC.Cyt.N.TP2_CBP_Bd.Flux)
+EC.Cyt.N.PGRB_NCOA_AC.Flux;
        EC.Cyt.N.TLGVYSXTLN.Concentration = EC.Cyt.N.TLGVYSXTLN.Amount/EC.Cyt.N.Volume;
        when (t=t.min) EC.Cyt.N.TLGVYSXTLN.Amount =
EC.Cyt.N.TLGVYSXTLN.InitialConcentration*EC.Cyt.N.Volume;
        EC.Cyt.N.TLGVYSXTLN.Amount:t = -EC.Cyt.N.TC3_BRG_Bd.Flux;
        real EC.SHBG.Amount(t) umol;
        real EC.SHBG.Concentration(t) uM;
        real EC.SHBG.InitialConcentration uM;
        real EC.SHBG_ES_Bd.Flux(t) umol/sec;
        real EC.SHBG_ES_Bd.Kf 1/(uM*sec);
        real EC.SHBG_ES_Bd.Kr 1/sec;
        EC.SHBG_ES_Bd.Kf = 1;
        EC.SHBG_ES_Bd.Flux = (((EC.SHBG_ES_Bd.Kf*EC.SHBG.Concentration)
*EC.ES.Concentration)-(EC.SHBG_ES_Bd.Kr*EC.SHBG_ES.Concentration))*EC.Vec;
```

```
        EC.SHBG_ES_Bd.Kr = 6.8E-4;
        real EC.SHBG_PG_Bd.Flux(t) umol/sec;
        real EC.SHBG_PG_Bd.Kf 1/(uM*sec);
        real EC.SHBG_PG_Bd.Kr 1/sec;
        EC.SHBG_PG_Bd.Kf = 10;
        EC.SHBG_PG_Bd.Flux = (((EC.SHBG_PG_Bd.Kf*EC.SHBG.Concentration)
*EC.PG.Concentration)-(EC.SHBG_PG_Bd.Kr*EC.SHBG_PG.Concentration))*EC.Vec;
        EC.SHBG_PG_Bd.Kr = 9.0E-5;
        EC.Vec = 2;
        EC.Volume = 2;
        EC.PG.Concentration = 0.0032;
        EC.SHBG_ES.InitialConcentration = 0;
        EC.ES.Concentration = 0.003671;
        EC.SHBG_PG.InitialConcentration = 0;
        EC.SHBG.InitialConcentration = 1.11;
        EC.Cyt.ESRc_mRNA.Concentration = EC.Cyt.ESRc_mRNA.Amount/EC.Cyt.Volume;
        when (t=t.min) EC.Cyt.ESRc_mRNA.Amount =
EC.Cyt.ESRc_mRNA.InitialConcentration*EC.Cyt.Volume;
        EC.Cyt.ESRc_mRNA.Amount:t = (EC.Cyt.mRNA_ESRn_Tr.Flux-EC.Cyt.ESR_Trl.Flux)-
EC.Cyt.mRNA_ESRc_Dgr.Flux;
        EC.Cyt.VQDPIGKQWC.Concentration = EC.Cyt.VQDPIGKQWC.Amount/EC.Cyt.Volume;
        when (t=t.min) EC.Cyt.VQDPIGKQWC.Amount =
EC.Cyt.VQDPIGKQWC.InitialConcentration*EC.Cyt.Volume;
        EC.Cyt.VQDPIGKQWC.Amount:t = EC.Cyt.C02_ESR_Dis.Flux-EC.Cyt.ESR1_ES_Bd.Flux;
        EC.Cyt.BDCIKSYKJY.Concentration = EC.Cyt.BDCIKSYKJY.Amount/EC.Cyt.Volume;
        when (t=t.min) EC.Cyt.BDCIKSYKJY.Amount =
EC.Cyt.BDCIKSYKJY.InitialConcentration*EC.Cyt.Volume;
        EC.Cyt.BDCIKSYKJY.Amount:t = (-EC.Cyt.C05_PGRA_Dis.Flux)
+EC.Cyt.PGRA_C01_Bd.Flux;
        EC.Cyt.NFKBIAc.Concentration = EC.Cyt.NFKBIAc.Amount/EC.Cyt.Volume;
        when (t=t.min) EC.Cyt.NFKBIAc.Amount =
EC.Cyt.NFKBIAc.InitialConcentration*EC.Cyt.Volume;
        EC.Cyt.NFKBIAc.Amount:t = ((((-EC.Cyt.NFKBIAc_Tr.Flux)+EC.Cyt.NFKBIA_Trl.Flux)-
EC.Cyt.NFKBIAc_3_Dgr.Flux)-EC.Cyt.NFKBIAc_NFKB1c_Bd.Flux)-EC.Cyt.CHUK_p_NFKBIAc_Bd.Flux;
        EC.Cyt.TJMSEORQAV.Concentration = EC.Cyt.TJMSEORQAV.Amount/EC.Cyt.Volume;
        when (t=t.min) EC.Cyt.TJMSEORQAV.Amount =
EC.Cyt.TJMSEORQAV.InitialConcentration*EC.Cyt.Volume;
        EC.Cyt.TJMSEORQAV.Amount:t = (-EC.Cyt.PGRA_Tr.Flux)+EC.Cyt.PGRA_PG_Bd.Flux;
        EC.Cyt.WSQTUBSXTX.Amount = EC.Cyt.WSQTUBSXTX.Concentration*EC.Cyt.Volume;
        EC.Cyt.TBJMXSSCJU.Concentration = EC.Cyt.TBJMXSSCJU.Amount/EC.Cyt.Volume;
        when (t=t.min) EC.Cyt.TBJMXSSCJU.Amount =
EC.Cyt.TBJMXSSCJU.InitialConcentration*EC.Cyt.Volume;
        EC.Cyt.TBJMXSSCJU.Amount:t = EC.Cyt.PGRA_PG_Bd.Flux-EC.Cyt.hsp90_3_Rgn.Flux;
        EC.Cyt.YINGPPRWIO.Concentration = EC.Cyt.YINGPPRWIO.Amount/EC.Cyt.Volume;
        when (t=t.min) EC.Cyt.YINGPPRWIO.Amount =
EC.Cyt.YINGPPRWIO.InitialConcentration*EC.Cyt.Volume;
        EC.Cyt.YINGPPRWIO.Amount:t = EC.Cyt.ESR1_ES_Bd.Flux-EC.Cyt.ESR1_Tr.Flux;
        EC.Cyt.VUVKKIUMSM.Concentration = EC.Cyt.VUVKKIUMSM.Amount/EC.Cyt.Volume;
        when (t=t.min) EC.Cyt.VUVKKIUMSM.Amount =
EC.Cyt.VUVKKIUMSM.InitialConcentration*EC.Cyt.Volume;
        EC.Cyt.VUVKKIUMSM.Amount:t = (-EC.Cyt.hsp90_1_Rgn.Flux)+EC.Cyt.ESR1_ES_Bd.Flux;
        EC.Cyt.RFFXXGONQA.Concentration = EC.Cyt.RFFXXGONQA.Amount/EC.Cyt.Volume;
        when (t=t.min) EC.Cyt.RFFXXGONQA.Amount =
EC.Cyt.RFFXXGONQA.InitialConcentration*EC.Cyt.Volume;
        EC.Cyt.RFFXXGONQA.Amount:t = (-EC.Cyt.PGRB_Frm.Flux)+EC.Cyt.PGRB_Trl.Flux;
        EC.Cyt.FCMRIEXQWG.Concentration = EC.Cyt.FCMRIEXQWG.Amount/EC.Cyt.Volume;
        when (t=t.min) EC.Cyt.FCMRIEXQWG.Amount =
EC.Cyt.FCMRIEXQWG.InitialConcentration*EC.Cyt.Volume;
        EC.Cyt.FCMRIEXQWG.Amount:t = EC.Cyt.PGRB_PG_Bd.Flux-EC.Cyt.PGRB_Tr.Flux;
        EC.Cyt.CHUK_p.Concentration = EC.Cyt.CHUK_p.Amount/EC.Cyt.Volume;
        when (t=t.min) EC.Cyt.CHUK_p.Amount =
EC.Cyt.CHUK_p.InitialConcentration*EC.Cyt.Volume;
        EC.Cyt.CHUK_p.Amount:t = ((((-EC.Cyt.CHUK_p_Dgr.Flux)
+EC.Cyt.NFKBIAc_2_Dgr.Flux)-EC.Cyt.CHUK_p_NFKBIAc_NFKB1c_1_Bd.Flux)
```

```
+EC.Cyt.NFKBIAc_1_Dgr.Flux)-EC.Cyt.CHUK_p_NFKBIAc_Bd.Flux;
        EC.Cyt.ADP.Concentration = EC.Cyt.ADP.Amount/EC.Cyt.Volume;
        when (t=t.min) EC.Cyt.ADP.Amount =
EC.Cyt.ADP.InitialConcentration*EC.Cyt.Volume;
        EC.Cyt.ADP.Amount:t = (EC.Cyt.CO5_PGRA_Dis.Flux+EC.Cyt.C02_ESR_Dis.Flux)
+EC.Cyt.C04_PGRB_Dis.Flux;
        EC.Cyt.SMMYREUOEE.Concentration = EC.Cyt.SMMYREUOEE.Amount/EC.Cyt.Volume;
        when (t=t.min) EC.Cyt.SMMYREUOEE.Amount =
EC.Cyt.SMMYREUOEE.InitialConcentration*EC.Cyt.Volume;
        EC.Cyt.SMMYREUOEE.Amount:t = (-EC.Cyt.CO_Hsp90_Bd.Flux)+EC.Cyt.Hsp90_Dis.Flux;
        EC.Cyt.NFKB1c.Concentration = EC.Cyt.NFKB1c.Amount/EC.Cyt.Volume;
        when (t=t.min) EC.Cyt.NFKB1c.Amount =
EC.Cyt.NFKB1c.InitialConcentration*EC.Cyt.Volume;
        EC.Cyt.NFKB1c.Amount:t = ((((-EC.Cyt.NFKB1c_Tr.Flux)+EC.Cyt.NFKBIAc_1_Dgr.Flux)
+EC.Cyt.NFKBIAc_Dgr.Flux)-EC.Cyt.NFKBIAc_NFKB1c_Bd.Flux)-
EC.Cyt.CHUK_p_NFKBIAc_NFKB1c_2_Bd.Flux;
        EC.Cyt.MEUHHCXDNY.Concentration = EC.Cyt.MEUHHCXDNY.Amount/EC.Cyt.Volume;
        when (t=t.min) EC.Cyt.MEUHHCXDNY.Amount =
EC.Cyt.MEUHHCXDNY.InitialConcentration*EC.Cyt.Volume;
        EC.Cyt.MEUHHCXDNY.Amount:t = (((-EC.Cyt.CHUK_p_NFKBIAc_NFKB1c_1_Bd.Flux)-
EC.Cyt.NFKBIAc_Dgr.Flux)+EC.Cyt.NFKBIAc_NFKB1c_Bd.Flux)+EC.Cyt.N.NFKBIAn_NFKB1n_Tr.Flux;
        EC.Cyt.PGRB.Concentration = EC.Cyt.PGRB.Amount/EC.Cyt.Volume;
        when (t=t.min) EC.Cyt.PGRB.Amount =
EC.Cyt.PGRB.InitialConcentration*EC.Cyt.Volume;
        EC.Cyt.PGRB.Amount:t = (EC.Cyt.PGRB_Frm.Flux-EC.Cyt.PGRB_hsp40_Bd.Flux)-
EC.Cyt.PGRB_Dgr.Flux;
        EC.Cyt.XKVYWVGFII.Concentration = EC.Cyt.XKVYWVGFII.Amount/EC.Cyt.Volume;
        when (t=t.min) EC.Cyt.XKVYWVGFII.Amount =
EC.Cyt.XKVYWVGFII.InitialConcentration*EC.Cyt.Volume;
        EC.Cyt.XKVYWVGFII.Amount:t = (-EC.Cyt.PGRA_Frm.Flux)+EC.Cyt.PGRA_Trl.Flux;
        EC.Cyt.KMXEIYOWMQ.Concentration = EC.Cyt.KMXEIYOWMQ.Amount/EC.Cyt.Volume;
        when (t=t.min) EC.Cyt.KMXEIYOWMQ.Amount =
EC.Cyt.KMXEIYOWMQ.InitialConcentration*EC.Cyt.Volume;
        EC.Cyt.KMXEIYOWMQ.Amount:t = (-EC.Cyt.CO_Hsp90_Bd.Flux)+EC.Cyt.CO_ATP_Bd.Flux;
        EC.Cyt.IVPLRKCCWI.Concentration = EC.Cyt.IVPLRKCCWI.Amount/EC.Cyt.Volume;
        when (t=t.min) EC.Cyt.IVPLRKCCWI.Amount =
EC.Cyt.IVPLRKCCWI.InitialConcentration*EC.Cyt.Volume;
        EC.Cyt.IVPLRKCCWI.Amount:t = EC.Cyt.C02_ESR_Dis.Flux-EC.Cyt.CO_1_Rgn.Flux;
        EC.Cyt.LBXTYHSEFK.Concentration = EC.Cyt.LBXTYHSEFK.Amount/EC.Cyt.Volume;
        when (t=t.min) EC.Cyt.LBXTYHSEFK.Amount =
EC.Cyt.LBXTYHSEFK.InitialConcentration*EC.Cyt.Volume;
        EC.Cyt.LBXTYHSEFK.Amount:t = (-EC.Cyt.CO_2_Rgn.Flux)+EC.Cyt.C04_PGRB_Dis.Flux;
        EC.Cyt.SFKBLJATXG.Concentration = EC.Cyt.SFKBLJATXG.Amount/EC.Cyt.Volume;
        when (t=t.min) EC.Cyt.SFKBLJATXG.Amount =
EC.Cyt.SFKBLJATXG.InitialConcentration*EC.Cyt.Volume;
        EC.Cyt.SFKBLJATXG.Amount:t = ((-EC.Cyt.NFKBIAc_2_Dgr.Flux)-
EC.Cyt.CHUK_p_NFKBIAc_NFKB1c_2_Bd.Flux)+EC.Cyt.CHUK_p_NFKBIAc_Bd.Flux;
        EC.Cyt.PGRBc_mRNA.Concentration = EC.Cyt.PGRBc_mRNA.Amount/EC.Cyt.Volume;
        when (t=t.min) EC.Cyt.PGRBc_mRNA.Amount =
EC.Cyt.PGRBc_mRNA.InitialConcentration*EC.Cyt.Volume;
        EC.Cyt.PGRBc_mRNA.Amount:t = ((-EC.Cyt.PGRBmRNAc_Dgr.Flux)-EC.Cyt.PGRB_Trl.Flux)
+EC.Cyt.PGRBmRNA_Tr.Flux;
        EC.Cyt.PGRA.Concentration = EC.Cyt.PGRA.Amount/EC.Cyt.Volume;
        when (t=t.min) EC.Cyt.PGRA.Amount =
EC.Cyt.PGRA.InitialConcentration*EC.Cyt.Volume;
        EC.Cyt.PGRA.Amount:t = (EC.Cyt.PGRA_Frm.Flux-EC.Cyt.PGRA_hsp40_Bd.Flux)-
EC.Cyt.PGRA_Dgr.Flux;
        EC.Cyt.VXLSQVSTNA.Concentration = EC.Cyt.VXLSQVSTNA.Amount/EC.Cyt.Volume;
        when (t=t.min) EC.Cyt.VXLSQVSTNA.Amount =
EC.Cyt.VXLSQVSTNA.InitialConcentration*EC.Cyt.Volume;
        EC.Cyt.VXLSQVSTNA.Amount:t = (-EC.Cyt.C04_PGRB_Dis.Flux)
+EC.Cyt.PGRB_CO1_Bd.Flux;
        EC.Cyt.HSP90AA1_pool.Concentration = EC.Cyt.HSP90AA1_pool.Amount/EC.Cyt.Volume;
        when (t=t.min) EC.Cyt.HSP90AA1_pool.Amount =
```

```
EC.Cyt.HSP90AA1_pool.InitialConcentration*EC.Cyt.Volume;
        EC.Cyt.HSP90AA1_pool.Amount:t = ((EC.Cyt.hsp90_1_Rgn.Flux
+EC.Cyt.hsp90_2_Rgn.Flux)-EC.Cyt.Hsp90_Dis.Flux)+EC.Cyt.hsp90_3_Rgn.Flux;
        EC.Cyt.EFUYJJRUFC.Concentration = EC.Cyt.EFUYJJRUFC.Amount/EC.Cyt.Volume;
        when (t=t.min) EC.Cyt.EFUYJJRUFC.Amount =
EC.Cyt.EFUYJJRUFC.InitialConcentration*EC.Cyt.Volume;
        EC.Cyt.EFUYJJRUFC.Amount:t = (-EC.Cyt.ESR1_ES_Bd.Flux)+EC.Cyt.ES_Tr.Flux;
        EC.Cyt.BUKQBUVSPL.Concentration = EC.Cyt.BUKQBUVSPL.Amount/EC.Cyt.Volume;
        when (t=t.min) EC.Cyt.BUKQBUVSPL.Amount =
EC.Cyt.BUKQBUVSPL.InitialConcentration*EC.Cyt.Volume;
        EC.Cyt.BUKQBUVSPL.Amount:t = ((EC.Cyt.CO_2_Rgn.Flux-EC.Cyt.PGRB_hsp40_Bd.Flux)-
EC.Cyt.PGRA_hsp40_Bd.Flux)+EC.Cyt.CO_3_Rgn.Flux;
        EC.Cyt.IBUAUBLBAJ.Concentration = EC.Cyt.IBUAUBLBAJ.Amount/EC.Cyt.Volume;
        when (t=t.min) EC.Cyt.IBUAUBLBAJ.Amount =
EC.Cyt.IBUAUBLBAJ.InitialConcentration*EC.Cyt.Volume;
        EC.Cyt.IBUAUBLBAJ.Amount:t = (-EC.Cyt.PGRB_PG_Bd.Flux)+EC.Cyt.C04_PGRB_Dis.Flux;
        EC.Cyt.QQDSAXKYSH.Concentration = EC.Cyt.QQDSAXKYSH.Amount/EC.Cyt.Volume;
        when (t=t.min) EC.Cyt.QQDSAXKYSH.Amount =
EC.Cyt.QQDSAXKYSH.InitialConcentration*EC.Cyt.Volume;
        EC.Cyt.QQDSAXKYSH.Amount:t = (EC.Cyt.CHUK_p_NFKBIAc_NFKB1c_1_Bd.Flux-
EC.Cyt.NFKBIAc_1_Dgr.Flux)+EC.Cyt.CHUK_p_NFKBIAc_NFKB1c_2_Bd.Flux;
        EC.Cyt.ATP.Concentration = EC.Cyt.ATP.Amount/EC.Cyt.Volume;
        when (t=t.min) EC.Cyt.ATP.Amount =
EC.Cyt.ATP.InitialConcentration*EC.Cyt.Volume;
        EC.Cyt.ATP.Amount:t = -EC.Cyt.CO_ATP_Bd.Flux;
        EC.Cyt.JWYDCVVNFY.Concentration = EC.Cyt.JWYDCVVNFY.Amount/EC.Cyt.Volume;
        when (t=t.min) EC.Cyt.JWYDCVVNFY.Amount =
EC.Cyt.JWYDCVVNFY.InitialConcentration*EC.Cyt.Volume;
        EC.Cyt.JWYDCVVNFY.Amount:t = EC.Cyt.C05_PGRA_Dis.Flux-EC.Cyt.PGRA_PG_Bd.Flux;
        EC.Cyt.ESR1.Concentration = EC.Cyt.ESR1.Amount/EC.Cyt.Volume;
        when (t=t.min) EC.Cyt.ESR1.Amount =
EC.Cyt.ESR1.InitialConcentration*EC.Cyt.Volume;
        EC.Cyt.ESR1.Amount:t = (EC.Cyt.ESR_Frm.Flux-EC.Cyt.ESR_Dgr.Flux)-
EC.Cyt.ESR1_CO1_Bd.Flux;
        EC.Cyt.IHYPKQPWTX.Concentration = EC.Cyt.IHYPKQPWTX.Amount/EC.Cyt.Volume;
        when (t=t.min) EC.Cyt.IHYPKQPWTX.Amount =
EC.Cyt.IHYPKQPWTX.InitialConcentration*EC.Cyt.Volume;
        EC.Cyt.IHYPKQPWTX.Amount:t = (((-EC.Cyt.NFKBIAc_p_Dgr.Flux)
+EC.Cyt.NFKBIAc_2_Dgr.Flux)+EC.Cyt.NFKBIAc_1_Dgr.Flux)+EC.Cyt.NFKBIAc_Dgr.Flux;
        EC.Cyt.GVEAOFSCUE.Concentration = EC.Cyt.GVEAOFSCUE.Amount/EC.Cyt.Volume;
        when (t=t.min) EC.Cyt.GVEAOFSCUE.Amount =
EC.Cyt.GVEAOFSCUE.InitialConcentration*EC.Cyt.Volume;
        EC.Cyt.GVEAOFSCUE.Amount:t = EC.Cyt.PGRB_PG_Bd.Flux-EC.Cyt.hsp90_2_Rgn.Flux;
        EC.Cyt.PGRAc_mRNA.Concentration = EC.Cyt.PGRAc_mRNA.Amount/EC.Cyt.Volume;
        when (t=t.min) EC.Cyt.PGRAc_mRNA.Amount =
EC.Cyt.PGRAc_mRNA.InitialConcentration*EC.Cyt.Volume;
        EC.Cyt.PGRAc_mRNA.Amount:t = ((-EC.Cyt.PGRA_Trl.Flux)+EC.Cyt.PGRAmRNA_Tr.Flux)-
EC.Cyt.PGRmRNAc_Dgr.Flux;
        EC.Cyt.VESHECAPCO.Concentration = EC.Cyt.VESHECAPCO.Amount/EC.Cyt.Volume;
        when (t=t.min) EC.Cyt.VESHECAPCO.Amount =
EC.Cyt.VESHECAPCO.InitialConcentration*EC.Cyt.Volume;
        EC.Cyt.VESHECAPCO.Amount:t = EC.Cyt.PGRB_hsp40_Bd.Flux-EC.Cyt.PGRB_CO1_Bd.Flux;
        EC.Cyt.CUUPSCJFCK.Concentration = EC.Cyt.CUUPSCJFCK.Amount/EC.Cyt.Volume;
        when (t=t.min) EC.Cyt.CUUPSCJFCK.Amount =
EC.Cyt.CUUPSCJFCK.InitialConcentration*EC.Cyt.Volume;
        EC.Cyt.CUUPSCJFCK.Amount:t = EC.Cyt.ESR_Trl.Flux-EC.Cyt.ESR_Frm.Flux;
        EC.Cyt.IHRRYRSKWQ.Concentration = EC.Cyt.IHRRYRSKWQ.Amount/EC.Cyt.Volume;
        when (t=t.min) EC.Cyt.IHRRYRSKWQ.Amount =
EC.Cyt.IHRRYRSKWQ.InitialConcentration*EC.Cyt.Volume;
        EC.Cyt.IHRRYRSKWQ.Amount:t = EC.Cyt.PGRA_hsp40_Bd.Flux-EC.Cyt.PGRA_CO1_Bd.Flux;
        EC.Cyt.CO.Concentration = EC.Cyt.CO.Amount/EC.Cyt.Volume;
        when (t=t.min) EC.Cyt.CO.Amount = EC.Cyt.CO.InitialConcentration*EC.Cyt.Volume;
        EC.Cyt.CO.Amount:t = ((EC.Cyt.CO_2_Rgn.Flux+EC.Cyt.CO_3_Rgn.Flux)
+EC.Cyt.CO_1_Rgn.Flux)-EC.Cyt.CO_ATP_Bd.Flux;
```

```
        EC.Cyt.VCKNCEABFO.Concentration = EC.Cyt.VCKNCEABFO.Amount/EC.Cyt.Volume;
        when (t=t.min) EC.Cyt.VCKNCEABFO.Amount =
EC.Cyt.VCKNCEABFO.InitialConcentration*EC.Cyt.Volume;
        EC.Cyt.VCKNCEABFO.Amount:t = EC.Cyt.CO5_PGRA_Dis.Flux-EC.Cyt.CO_3_Rgn.Flux;
        EC.Cyt.HXPQSSVMJO.Concentration = EC.Cyt.HXPQSSVMJO.Amount/EC.Cyt.Volume;
        when (t=t.min) EC.Cyt.HXPQSSVMJO.Amount =
EC.Cyt.HXPQSSVMJO.InitialConcentration*EC.Cyt.Volume;
        EC.Cyt.HXPQSSVMJO.Amount:t = ((-EC.Cyt.PGRB_PG_Bd.Flux)+EC.Cyt.PG_Tr.Flux)-
EC.Cyt.PGRA_PG_Bd.Flux;
        EC.Cyt.IIYBUWADRK.Concentration = EC.Cyt.IIYBUWADRK.Amount/EC.Cyt.Volume;
        when (t=t.min) EC.Cyt.IIYBUWADRK.Amount =
EC.Cyt.IIYBUWADRK.InitialConcentration*EC.Cyt.Volume;
        EC.Cyt.IIYBUWADRK.Amount:t = (-EC.Cyt.C02_ESR_Dis.Flux)+EC.Cyt.ESR1_CO1_Bd.Flux;
        EC.Cyt.JNGRHJTOEM.Concentration = EC.Cyt.JNGRHJTOEM.Amount/EC.Cyt.Volume;
        when (t=t.min) EC.Cyt.JNGRHJTOEM.Amount =
EC.Cyt.JNGRHJTOEM.InitialConcentration*EC.Cyt.Volume;
        EC.Cyt.JNGRHJTOEM.Amount:t = ((EC.Cyt.CO_Hsp90_Bd.Flux-EC.Cyt.PGRA_CO1_Bd.Flux)-
EC.Cyt.ESR1_CO1_Bd.Flux)-EC.Cyt.PGRB_CO1_Bd.Flux;
        PRA_PRB_Ratio = EC.Cyt.PGRA.Concentration/EC.Cyt.PGRB.Concentration;
        EC.PG.Amount = EC.PG.Concentration*EC.Volume;
        EC.SHBG_ES.Concentration = EC.SHBG_ES.Amount/EC.Volume;
        when (t=t.min) EC.SHBG_ES.Amount = EC.SHBG_ES.InitialConcentration*EC.Volume;
        EC.SHBG_ES.Amount:t = EC.SHBG_ES_Bd.Flux;
        EC.ES.Amount = EC.ES.Concentration*EC.Volume;
        EC.SHBG_PG.Concentration = EC.SHBG_PG.Amount/EC.Volume;
        when (t=t.min) EC.SHBG_PG.Amount = EC.SHBG_PG.InitialConcentration*EC.Volume;
        EC.SHBG_PG.Amount:t = EC.SHBG_PG_Bd.Flux;
        EC.SHBG.Concentration = EC.SHBG.Amount/EC.Volume;
        when (t=t.min) EC.SHBG.Amount = EC.SHBG.InitialConcentration*EC.Volume;
        EC.SHBG.Amount:t = (-EC.SHBG_ES_Bd.Flux)-EC.SHBG_PG_Bd.Flux;
}
```
